# Analysis of muntjac deer genome and chromatin architecture reveals rapid karyotype evolution

**DOI:** 10.1038/s42003-020-1096-9

**Published:** 2020-09-01

**Authors:** Austin B. Mudd, Jessen V. Bredeson, Rachel Baum, Dirk Hockemeyer, Daniel S. Rokhsar

**Affiliations:** 1 Department of Molecular and Cell Biology, University of California, Berkeley, CA USA; 2 Innovative Genomics Institute, University of California, Berkeley, CA USA; 3 Chan Zuckerberg Biohub, San Francisco, CA USA; 4 Department of Energy Joint Genome Institute, Walnut Creek, CA USA; 5 Molecular Genetics Unit, Okinawa Institute of Science and Technology Graduate University, Onna, Okinawa Japan

**Keywords:** Comparative genomics, Genome evolution, Evolutionary genetics

## Abstract

Closely related muntjac deer show striking karyotype differences. Here we describe chromosome-scale genome assemblies for Chinese and Indian muntjacs, *Muntiacus reevesi* (2*n* = 46) and *Muntiacus muntjak vaginalis* (2*n* = 6/7), and analyze their evolution and architecture. The genomes show extensive collinearity with each other and with other deer and cattle. We identified numerous fusion events unique to and shared by muntjacs relative to the cervid ancestor, confirming many cytogenetic observations with genome sequence. One of these *M. muntjak* fusions reversed an earlier fission in the cervid lineage. Comparative Hi-C analysis showed that the chromosome fusions on the *M. muntjak* lineage altered long-range, three-dimensional chromosome organization relative to *M. reevesi* in interphase nuclei including A/B compartment structure. This reshaping of multi-megabase contacts occurred without notable change in local chromatin compaction, even near fusion sites. A few genes involved in chromosome maintenance show evidence for rapid evolution, possibly associated with the dramatic changes in karyotype.

## Abstract (web summary)

Mudd et al. provide chromosome-scale genome assemblies for the Chinese and Indian muntjac deer and demonstrate evidence for rapid evolution of genes involved in chromosome maintenance. Their comparative analyses generate new insights into rapid karyotype evolution.

## Introduction

One of the most spectacular examples of rapid karyotype evolution is found in the muntjacs, a genus of small Asian deer whose karyotypes vary from 2*n* = 6 in the female Indian muntjac *Muntiacus muntjak vaginalis*, the smallest known chromosome number of any mammal^1^, to 2*n* = 46 in the Chinese muntjac *Muntiacus reevesi*^2^. Since the discovery of the *M. muntjak* karyotype, cytogeneticists have explored the mechanism of chromosome variation in this lineage. The *M. reevesi* karyotype generally resembles those of other deer and cattle^3^, implying rapid reduction of chromosome number on the *M. muntjak* lineage. Hsu et al.^4^ proposed that this reduction occurred through multiple tandem and centric fusions from an *M. reevesi*-like ancestor, a finding supported by Liming et al.^5^ Fusions were further implicated by the discovery of interstitial centromeric satellites from *M. reevesi* embedded in *M. muntjak* chromosomes^6^ along with interstitial telomeric sequences^7,8^.

The application of chromosome painting techniques by Yang et al.^9^ and cosmid clone fluorescence in situ hybridization (FISH) by Frönicke et al.^10^ provided direct molecular cytogenetic evidence for the fusion theory. Soon afterwards, the 2*n* = 46 *M. reevesi* karyotype was found to contain independent fusions not shared with *M. muntjak*^11^, and Yang et al.^11^ concluded that multiple tandem and centric fusions must have occurred independently in the *M. muntjak* and *M. reevesi* lineages, a finding supported by phylogenetics^12^. Following this discovery, Chi et al. traced the changes in the muntjac karyotypes using chromosome painting between *M. reevesi* and other pecorans as well as bacterial artificial chromosomes (BACs) mapped by FISH between *M. muntjak* and *M. reevesi*^3,13^. As further support for the tandem fusion theory, several sequence-based studies have found evidence for the juxtaposition of centromeric repeats and telomeric sequences at fusion sites^14–16^.

Building on these pioneering cytogenetic efforts, we set out to explore muntjac karyotype evolution using genome sequence comparisons. To this end, we produced the first chromosome-scale assemblies of both *M. muntjak* and *M. reevesi*, described below, with contiguity metrics that surpass those of earlier draft assemblies^17,18^. For comparative purposes, we leveraged published chromosome-scale assemblies of *Bos taurus* (cow)^19^ and *Cervus elaphus* (red deer)^20^ as well as a sub-chromosome assembly of *Rangifer tarandus* (reindeer)^21^ to map karyotype changes across the cervid lineage. From this analysis, we determined the number, distribution, and timing of shared and lineage-specific fusion events, corroborating prior molecular cytogenetic findings and extending them to nucleotide resolution. Surprisingly, we noticed that one fusion event in the *M. muntjak* lineage reversed a chromosome fission that had occurred earlier in the ancestral cervid lineage. In another case, we found a pair of ancestral cervid chromosomes that likely fused independently in the *M. muntjak* and *M. reevesi* lineages.

Chromosome-scale analyses provide new genomic insights into the unique evolutionary history of these two karyotypically divergent species. The muntjac chromosomes show extensive collinearity with each other and with red deer and cow, demonstrating that the chromosome fusions occurred without disrupting gene order. This phenomenon is therefore distinct from the extensive rearrangements found in cancer due to chromothripsis^22^. Despite the high degree of collinearity, we found that chromosome fusions in the muntjacs altered long-range, three-dimensional genome organization in interphase nuclei including A/B compartment structure, although the impact of these changes on gene regulation and chromosome maintenance is unclear. While the molecular mechanism driving rapid karyotype change in muntjacs is not yet known, comparison of nearly 20,000 gene orthologs between the two species identified a number of genes with accelerated evolution in muntjacs, several of which are plausibly associated with chromosome maintenance and are therefore candidates for further study.

## Results and discussion

### Assembly and annotation

To investigate the tempo and mode of muntjac chromosome evolution, we generated high-quality, chromosome-scale genome assemblies for *M. muntjak* and *M. reevesi* (Supplementary Table 1) using a combination of linked reads^23^ (10x Genomics Chromium Genome) and chromatin conformation capture^24^ (Dovetail Genomics Hi-C; Supplementary Table 2, Methods). The resulting assemblies each contain 2.5 Gb of contig sequence with contig N50 lengths over 200 kb (Supplementary Table 1). In both assemblies, over 92% of contig sequence is anchored to chromosomes. Compared with publicly available assemblies^17,18^, the assemblies described here represent a hundredfold improvement in scaffold N50 length and severalfold improvement in contig N50 length. As typical for short-read assemblies, our muntjac assemblies are largely complete with respect to genic sequences (see below) but likely underrepresent repetitive sequences such as pericentromeric heterochromatin and repetitive subtelomeric regions, precluding further analysis of the sequence at fusions sites. The standard for analyzing the tandem fusion sites at the sequence level therefore remains BAC sequences spanning fusion sites^15,16^, which reported proximity of centromeric and telomeric repetitive sequences as expected for head-tail fusions.

The assembled chromosome numbers recapitulate the karyotypes reported in the literature, 2*n* = 6 for female *M. muntjak*^1^ (Supplementary Fig. 1) and 2*n* = 46 for *M. reevesi*^2^ (Supplementary Fig. 2). *M. reevesi* chromosomes were validated against and numbered according to chromosome painting data from Chi et al.^3^ For *M. muntjak*, we aligned 377 previously sequenced BACs^15,16,25^ and, based on corresponding FISH location data^15,16,25^, found that 360 (95%) of BACs align to the expected chromosomes. Of the 17 BACs that align to a different chromosome than expected by FISH, 16 align to our assembly in regions of conserved collinearity among cow, red deer, and muntjac chromosomes. The high degree of conserved collinearity across these regions and throughout the genome supports the correctness of our assemblies and suggests that the FISH-based chromosome assignments of these BACs are likely errors. Only one of these 17 BACs aligns to two of our assembled *M. muntjak* chromosomes, indicating a possible local misassembly or BAC construction error.

**Fig. 1:**
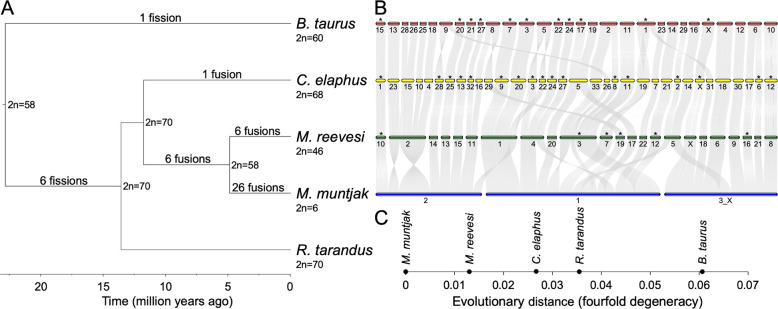
Evolutionary and phylogenomic analyses. **a** The phylogenetic tree of the five analyzed species, calculated from fourfold degenerate sites and divergence time confidence intervals, was visualized with FigTree (commit 901211e; https://github.com/rambaut/figtree). The ancestral karyotype at each node and the six branches with fission and fusion events relative to the ancestral karyotype were labeled on the tree. The lack of fissions or fusions on the *R. tarandus*-specific branch as well as the timings of the cervid-specific and *B. taurus*-specific fissions were derived from literature^30^. **b** The alignment plot was generated with jcvi.graphics.karyotype (v0.8.12; https://github.com/tanghaibao/jcvi) using runs of collinearity containing at least 25 kb of aligned sequence between *B. taurus*, *C. elaphus*, *M. reevesi*, and *M. muntjak*. *R. tarandus* was excluded, as it is not a chromosome-scale assembly. Chromosomes that have been inverted in this image relative to their original assembly orientations are marked with asterisks. **c** Pairwise distances in substitutions per fourfold degenerate site extracted from the RAxML (v8.2.11)^90^ phylogenetic tree using Newick utilities (v1.6)^87^ were shown relative to the reference genome *M. muntjak*.

For each muntjac genome, we annotated ~26,000 protein-coding genes based on homology with *B. taurus*^19^, *Ovis aries* (sheep)^26^, and *Homo sapiens* (human)^27^. Over 98% of these predicted genes could be functionally annotated by InterProScan (v5.34-73.0)^28^. We identified 19,649 one-to-one gene orthologs between the two muntjac species as well as 7,953 one-to-one gene orthologs present in the two muntjacs, *B. taurus*^19^, *C. elaphus*^20^, and *R. tarandus*^21^. These ortholog sets were used in the evolutionary and phylogenomic analyses below (Fig. 1a, c, Supplementary Table 3, Methods). Gene set comparisons (Supplementary Fig. 3) show that our muntjac annotations include several thousand more conserved pecoran genes than are found in the *C. elaphus* and *R. tarandus* annotations and demonstrate comparable completeness to *B. taurus*, supporting the accuracy of the muntjac assemblies in genic regions.

### Comparative analysis

In order to study sequence and karyotype evolution, we aligned the two muntjac assemblies to each other and to *B. taurus*^19^ as well as *B. taurus* to *C. elaphus*^20^ and *R. tarandus*^21^. The pairwise alignment of the muntjac genomes contains 2.45 Gb of contig sequence, or over 97% of the assembled contig sequence lengths. The average sequence identity of 98.5%, excluding indels, reflects the degree of sequence conservation between the two species and their recent divergence. In comparison, alignments of red deer, reindeer, and muntjacs to *B. taurus* contain 1.80–2.21 Gb of contig sequences with 92.7–93.2% average identity. Analysis of runs of collinear sequence identified breaks in synteny that, when projected onto the phylogeny, reveal the timing of fission and fusion events in each lineage. These analyses required that shared changes be present in the same order and orientation between species (Fig. 1a, b, Supplementary Fig. 4, Methods).

### Chromosome evolution

We assessed chromosome evolution in *M. muntjak* (MMU) and *M. reevesi* (MRE) using *B. taurus* (BTA) and *C. elaphus* (CEL) as outgroups. For the purposes of discussing chromosome dynamics across these species, it is convenient to use a common reference system. Since pecoran karyotypes exhibit broadly conserved syntenic units^3,29^, we used the well-characterized *B. taurus* as the primary reference and denote chromosome regions by their BTA chromosome identifiers in the text. Corresponding chromosomes or chromosome-scale units in other species can be easily traced in Fig. 1b and Supplementary Figs. 4–6. We corroborated prior reports in literature^30^ that:

1. In the last common ancestor of cervids and *B. taurus*, segments corresponding to the two cow chromosomes BTA26 and BTA28 were present as a single chromosome. This ancestral state, corresponding to BTA26_28, is retained in *C. elaphus* and the muntjacs.
2. Twelve chromosomes of the cervid ancestor arose by fission of metacentric or submetacentric chromosomes represented by six cow chromosomes (BTA1 → CEL19 and CEL31; BTA2 → CEL8 and CEL33; BTA5 → CEL3 and CEL22; BTA6 → CEL6 and CEL17; BTA8 → CEL16 and CEL29; and BTA9 → CEL26 and CEL28; Supplementary Table 4).
3. Although chromosomes homologous to BTA17 and BTA19 were fused in the *C. elaphus* lineage as CEL5, this fusion is unique to the *C. elaphus* lineage, and these cow chromosomes correspond to distinct ancestral cervid chromosomes.

In the muntjacs, we found six fusions shared by *M. muntjak* and *M. reevesi* (Supplementary Fig. 5; Supplementary Table 5): BTA7/BTA3, BTA5prox/BTA22, BTA2dist/BTA11, BTA18/BTA25/BTA26_28 (counting the fusion of three ancestral chromosomes as two fusion events), and BTA27/BTA8dist. All six of these fusions shared by *M. muntjak* and *M. reevesi* were also corroborated in previous BAC-FISH analyses of *Muntiacus crinifrons*, *Muntiacus feae*, and *Muntiacus gongshanensis*^31,32^.

After the divergence of *M. muntjak* and *M. reevesi*, each lineage experienced additional fusions. In the *M. reevesi* lineage, there were six fusions (Supplementary Table 6): BTA7_3/BTA5dist, BTA18_25_26_28/BTA13, BTA2prox/BTA9dist/BTA2dist_11, BTA5prox_22/BTA24, and BTA29/BTA16.

In the *M. muntjak* lineage, the three chromosomes arose via 26 lineage-specific fusions (Supplementary Table 7):

- MMU1: BTA7_3/BTA5prox_22/BTA17/BTA2prox/BTA1dist/BTA29/BTA8prox/BTA9dist/BTA19/BTA24/BTA23/BTA14/BTA2dist_11,
- MMU2: BTA15/BTA13/BTA18_25_26_28/BTA9prox/BTA20/BTA21/BTA27_8dist/BTA5dist, and
- MMU3: BTAX/BTA1prox/BTA4/BTA16/BTA12/BTA6prox/BTA6dist/BTA10.

While both *M. muntjak* and *M. reevesi* karyotypes include chromosomes that arose by fusion of BTA13 and BTA18_25_26_28, these events likely occurred independently. Consistent with our analysis, published BAC-FISH mapping of *M. reevesi* against *M. crinifrons*, *M. feae*, and *M. gongshanensis* found different locations of BTA13 and BTA18_25_26_28 in the muntjac species^31,32^, which support the conclusion that these were independent, lineage-specific fusion events.

In total, we found 38 fusion events and no fission events in the muntjac lineage (Fig. 1a). All of the *M. reevesi* fusions identified by our comparative analysis are substantiated by BAC-FISH from Frohlich et al.^30^, and all of the *M. muntjak* fusions are corroborated by the BAC-FISH findings of Chi et al.^13^

In order to examine the rates of karyotype change, we first estimated divergence times using our nuclear genome alignments (Methods). Our estimate of ~4.9 million years for the divergence of *M. muntjak* and *M. reevesi* (Fig. 1a, Supplementary Table 8) is consistent with recent estimates based on mitochondrial sequences^33,34^, with the identified proliferation of *Muntiacus spp*. in the Late Pliocene and Early Pleistocene^35^, and with dating of the oldest fossil attributed to the genus *Muntiacus*^36^ at ~8 million years ago. Another recent estimate of ~3.2 million years divergence between *M. muntjak* and *M. reevesi* based on nuclear genome alignments^18^ lies within our confidence interval. Similarly, estimates for the age of the last common cervid and bovid-cervid ancestors depend on the method and dataset but are in broad agreement (Supplementary Table 8).

From our calculated divergence times, we conservatively estimated that the rate of karyotype change in the *M. muntjak* lineage is an order of magnitude greater the mammalian average and is elevated, to a lesser extent, in the *M. reevesi* and stem muntjac lineages. During the ~4.9 million years since the divergence of *M. muntjak* and *M. reevesi*, the *M. muntjak* lineage experienced 26 fusions for a rate of ~5.3 changes per million years. Even allowing for the broad 95% confidence interval for the muntjac divergence of 2.9–6.5 million years (Supplementary Table 8)^37^, this rate is at least order of magnitude greater than the mammalian average of ~0.4 changes per million years estimated by Maruyama and Imai^38^ or ~0.36 changes per million years among artiodactyls estimated by Bush et al.^39^ To a lesser extent, the rates of change on the *M. reevesi* lineage (~1.2 changes per million years) and muntjac stem lineage (~0.87 changes per million years) also appear to be elevated compared with mammals. The nucleotide and temporal divergence between the two muntjac species (Fig. 1a, c, Supplementary Table 3) is comparable to the divergence between humans and chimpanzees^40,41^. The observed chromosome dynamism in muntjacs, however, far exceeds the rate in the chimpanzee and human lineages, which famously differ by just a single fusion on the human lineage^42^.

### Reversal of a cervid-specific fission in *M. muntjak*

While analyzing the fission and fusion events, we noticed that a fusion in *M. muntjak* appears to have reversed, to the resolution of our assembly, the cervid-specific fission of the ancestral chromosome corresponding to BTA6 (Supplementary Fig. 6). Although both the ancestral fission and *M. muntjak*-specific fusion have been noted individually in chromosome painting studies^3,13,43^, their apparent symmetric relationship has not been discussed. By taking advantage of the higher resolution of sequence comparisons relative to chromosome painting, we found that the segments orthologous to MRE16 and MRE21 are maintained in the same orientation in BTA6 and MMU3_X, indicating that the fusion in *M. muntjak* occurred at the same chromosome ends that were produced in the ancestral cervid fission. Alternately, it is possible that the fusion of MRE16 and MRE21 found in the clade of Indian, Gongshan, Fea’s and Black muntjacs represents an ancestral condition and that the existence of MRE16 and MRE21 as individual chromosomes in the Chinese muntjac and other deer is due to a convergent fission. This would, however, go against the general trend towards chromosome fusions in this lineage.

Given the high rate of fusion in *M. muntjak*, we considered the possibility that such a reversal could happen by chance. To this end, we simulated a simplified model for karyotype change with four rules: (1) only one fission is allowed per chromosome; (2) all fissions occur first, followed by all fusions; (3) for each fission, a chromosome is chosen at random; and (4) for each fusion, chromosomes and their relative orientations are chosen at random. From a starting karyotype of *n* = 29, representing the last common ancestor of cervids and *B. taurus*^30^, we simulated the model of fissions and fusions to 1 million iterations per fission-fusion combination (Supplementary Fig. 7). The *M. muntjak* lineage, with six fissions and 32 fusions, had a 4.1% probability of at least one fusion reversing a prior fission. In comparison, the *C. elaphus* lineage, with six fissions and one fusion, had only a 0.13% probability of reversal by chance, and the *M. reevesi* lineage, with six fissions and 12 fusions, had a 1.5% chance of reversal. Thus, even given the large number of fusions in muntjacs, the probability of a chance reversal of a previous fission is small. The reversal, however, could have been aided by unmodeled effects of differential chromosome fusion probability arising, for example, by chromosome proximity in the nucleus.

### Changes in three-dimensional genome structure after karyotype change

**Fig. 2:**
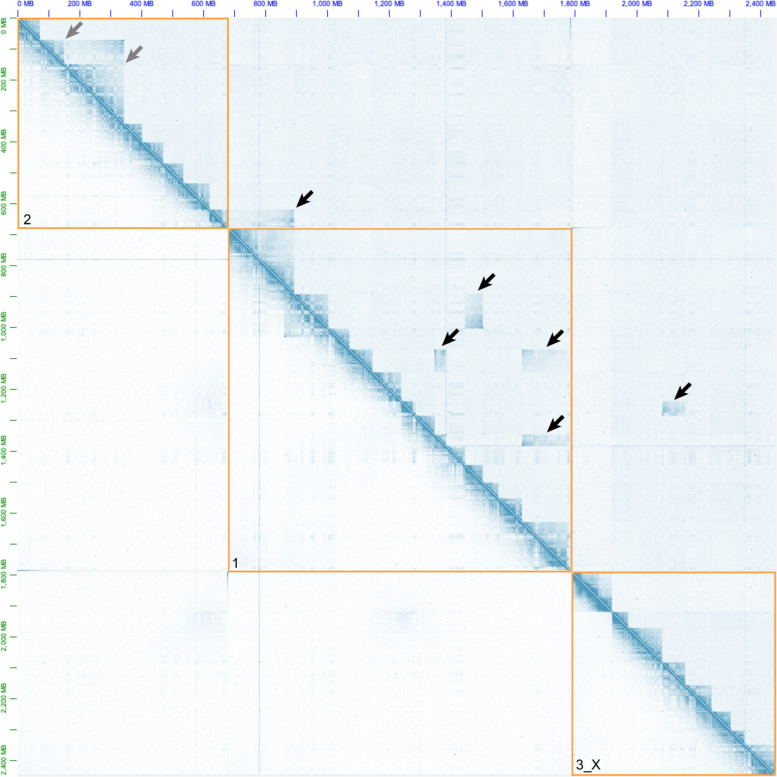
Chromosome Hi-C contact maps. Visualization of *M. muntjak* (below the diagonal) and *M. reevesi* (above the diagonal) Hi-C contact maps in Juicebox (v1.11.08)^62^ using the *M. muntjak* assembly as the reference. Orange boxes demarcate the boundaries of the three *M. muntjak* chromosomes, which are ordered as in Fig. 1. Chromosome numbers are provided in the lower-left corner of each. The intensity of blue pixels is proportional to the contact frequency between *x* and *y* pairs of genomic loci. The highest intensity pixels are along the diagonal of each chromosome, indicating a high degree of contacts between loci in close proximity. The checker board/striped patterns near the diagonal reflect fewer contacts between neighboring loci and increased contacts between more distant loci due to the three-dimensional chromatin folding (i.e., A/B compartment) structure within nuclei. In the upper triangle, the step-like pattern of high-density contacts along the diagonal is a result of conserved collinearity between *M. reevesi* and *M. muntjak* chromosomes; however, six blocks of high-frequency contacts (black arrows) can be observed off the diagonal and reflect large structural differences resulting from chromosome fission and fusion events. Two inverted segments (gray arrows) can also be observed.

Despite the extensive fusions documented above for *M. muntjak* and *M. reevesi*, the genomes are locally very similar, with 98.5% identity in aligned regions and a nucleotide divergence of 0.0130 substitutions per site, based on fourfold degenerate positions. Our chromatin conformation capture (Hi-C) data allowed us to examine the impact of these chromosome rearrangements on megabase (Mb) and longer length scales, as chromosome segments became juxtaposed in novel combinations. Focusing first on the *M. muntjak* and *M. reevesi* lineage-specific fusion sites (Supplementary Tables 4–7), we noted the maintenance of distinct Hi-C boundaries in several examples, such as the junction between the X and autosomal segments on MMU3_X circa 133 Mb. Other fusion sites, however, show no notable difference compared with the rest of the genome in *M. muntjak*. As expected, *M. reevesi* shows a clear distinction between intra- and inter-chromosome contacts, including across fusion sites in *M. muntjak* (Fig. 2). To quantify the chromatin changes at these fusion sites, we divided the genomes into 1 Mb bins and compared normalized inter-bin Hi-C contacts between bins 5 Mb apart in the two species, using the *M. muntjak* assembly as the backbone for comparison (Supplementary Fig. 8). Supporting the initial visual analysis, we found that most bins containing a fusion site have fewer long-range chromatin contacts in *M. reevesi* (averaging 0.16 ± 0.09 normalized contacts per bin) compared with *M. muntjak* (averaging 0.62 ± 0.35 normalized contacts per bin), although we identified bins with few contacts in both species (Supplementary Fig. 8).

**Fig. 3:**
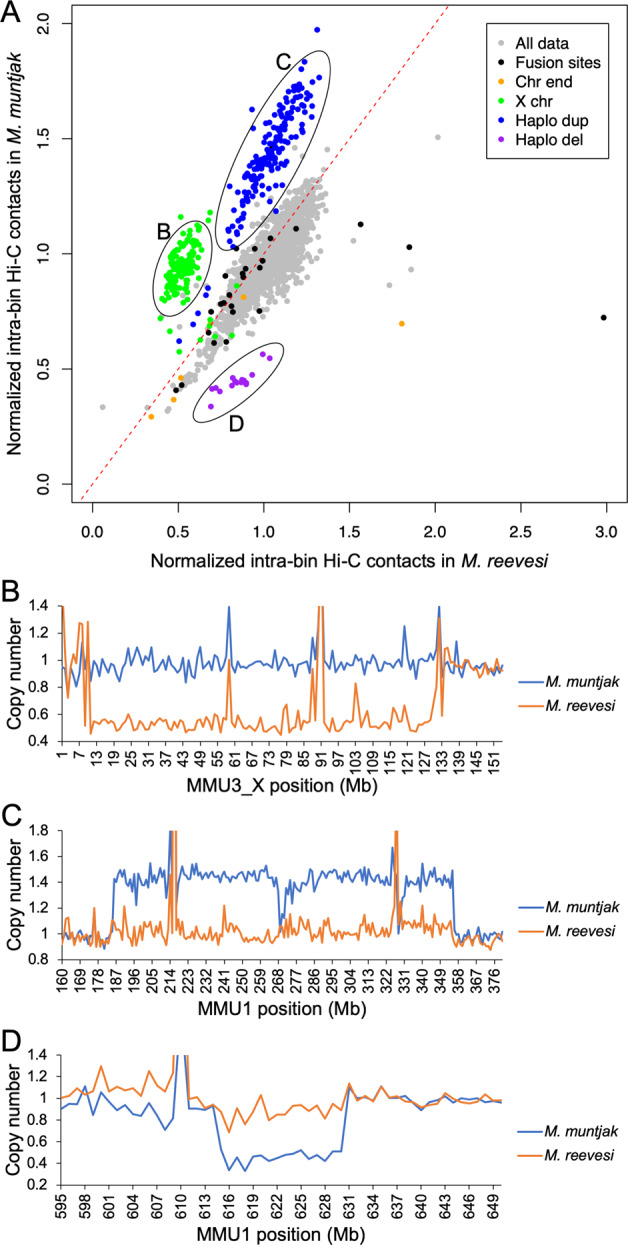
Evaluation of inter-chromosome contacts. **a** Normalized 1 Mb intra-bin Hi-C contacts for *M. muntjak* (*y*-axis) vs. *M. reevesi* (*x*-axis) with the bins containing the *M. muntjak* lineage-specific fusion sites (Supplementary Table 7), chromosome ends, the X chromosome, the potential *M. muntjak* haplotype-specific duplication, and the potential *M. muntjak* haplotype-specific deletion colored. The expected result of conserved Hi-C contacts was represented with a dashed red line. For fusion site ranges spanning two bins, the bin containing the majority of the fusion site range was deemed to be the fusion site bin. **b**–**d** Copy number was calculated from normalized coverage of adapter-trimmed 10x Genomics linked reads for three regions with variation in the chromatin contacts: **b** the X chromosome, **c** the potential *M. muntjak* haplotype-specific duplication, and **d** the potential *M. muntjak* haplotype-specific deletion, with the copy number of *M. muntjak* in blue and *M. reevesi* in orange.

In order to test whether differences are present at a more local level, we next compared normalized 1 Mb intra-bin Hi-C contacts between the two species, again using the *M. muntjak* assembly as the backbone for comparison. We found that most of the chromatin contacts are consistent between the two muntjacs, including all but three of the bins containing fusion sites (Fig. 3a, Supplementary Fig. 9). Several regions, however, show distinctive variation in chromatin contacts between the two species: the X chromosome and two regions on MMU1 (186–355 and 615–630 Mb). Since our sequenced *M. reevesi* sample was male^11^ while the sequenced *M. muntjak* sample was female^44^, we expected a difference in chromatin contacts on the X chromosome, a finding that was further supported by analysis of copy number across the genome using the 10x Genomics linked reads (Fig. 3b). From this copy number analysis, we also hypothesized that the two regions on MMU1 (186–355 and 615–630 Mb) represent a haplotype-specific duplication and a haplotype-specific deletion, respectively, which would explain the difference in chromatin signal between the two muntjac sequences (Fig. 3c, d). Since our sequencing data were generated from cell lines^11,44^, it is possible that these haplotype-specific differences could have arisen during cell culture. Further study is needed to confirm that these are bona fide segregating structural variants in *M. muntjak*. Nonetheless, although the inter-bin analysis identified long-range chromatin changes between sites 5 Mb apart, our quantitative comparison of 1 Mb intra-bin chromatin contacts found substantial chromatin conservation between the genome assemblies, including nearly all of the fusion sites. This conclusion is further supported by intra-bin analysis with 100 kb bins (Supplementary Fig. 10).

On a multi-megabase length scale, mammalian chromosomes can be subdivided into alternating A/B compartments based on intra-chromosome contacts; these compartments correspond to open and closed chromatin, respectively, and differ in gene density and GC content^24^. To test whether these compartments are conserved or disrupted by fusions, we computed the A/B chromatin compartment structures for *M. muntjak* and *M. reevesi* from the Hi-C data, again using the *M. muntjak* assembly as the backbone for comparison (Supplementary Fig. 11). We found that, in general, compartment boundaries are not well conserved between the muntjacs. Specifically, for A/B compartments larger than 3 Mb, only 17 compartments are completely conserved between the two species, out of 221 A/B compartments analyzed in *M. muntjak* and 161 in *M. reevesi*. We found that many of the compartments in *M. reevesi* are subdivided into multiple compartments in *M. muntjak*. Combining our analysis of A/B compartments and chromatin contacts, we found that the extensive set of fusions in the *M. muntjak* lineage altered its three-dimensional genome structure at the multi-megabase scale while still maintaining conservation at the local level. These large-scale chromatin changes that accompany karyotype change must have only limited effects on the underlying gene expression, since the two muntjac species can produce viable but sterile hybrid offspring^45^. Similar uncoupling between genome topology and gene expression has been observed in *Drosophila melanogaster*^46^.

### Genic evolution accompanying rapid karyotype change

Finally, we searched for genic differences between the two muntjacs that may have accompanied rapid karyotype evolution. These could, for example, be mutations that led to dysfunctional chromosome maintenance and thus triggered the rapid occurrence of multiple fusions, such as by destabilization of telomeres. More subtly, these genic changes could have occurred as a response to chromosome change. For example, the dramatic reduction in the number of telomeres following large-scale fusion could be permissive for mutations that make telomere maintenance less efficient. Our survey of evolutionary rates and gene family differences between muntjacs identified hundreds of candidates for further study (Supplementary Data 1). Although many genes in this list have no obvious relationship to chromosome biology, we found evidence for positive selection of centromere-associated proteins CENPQ and CENPV and meiotic double strand break protein MEI4 as well as expansion of the nucleosome-binding domain-containing HMG14 family in *M. muntjak*. Proteins encoded by these genes are central in DNA metabolism and chromosome biology, and mutations may have contributed to establishing a permissive cellular environment that allowed successive fusion events and the rapid evolution of muntjac karyotypes.

## Conclusions

Rapid karyotype evolution, often called karyotypic megaevolution^47^ or chromosomal tachytely^48^, has been found in various taxa, including rodents^49^, bears^50^, and gibbons^51^ and as a byproduct of chromosome instability in cancer^52^. Here, we present and analyze chromosome-scale genome assemblies of two muntjac deer whose karyotypes differ dramatically: the Indian muntjac *M. muntjak* (2*n* = 6) and the Chinese muntjac *M. reevesi* (2*n* = 46). Although many insights into muntjac genome evolution have been obtained through cytogenetic analysis as described in the introduction, the two chromosome-scale genome sequences reported here enable new genome-wide comparative analyses of intra-chromosome organization and gene evolution.

Our new muntjac genome assemblies took advantage of Hi-C sequencing to establish physical linkage at long distances. The longest *M. muntjak* chromosome, MMU1, is over a gigabase in length, yet our assembly correctly recapitulates organizational features identified by chromosome painting. Remarkably, Hi-C contacts are observed even across the extended pericentromeric region of MMU1, suggesting that this repetitive sequence is relatively compact in interphase nuclei. The independent corroboration of the global structure of our assembly by cytogenetic data demonstrates that Hi-C-based chromosome assembly is a robust method that, in the future, could be used for other genomes with large chromosomes, such as salamanders^53^ and conifers^54^. The demonstration of collinearity between the muntjac genomes and relative to cervid and cow chromosomes provides further support for the accuracy of Hi-C-based chromosome assembly.

Comparative analysis of the genome sequences of muntjacs, red deer, and cow both confirms the evolutionary sequence of fissions and fusions described cytogenetically and expands upon this prior work. We found that chromosome segments in cervids and cow have remained highly collinear since their divergence ~20 million years ago, aside from the discrete fission and fusion events shown in Fig. 1a, b. This, in turn, implies that the translocations and fusions observed in the muntjacs were not accompanied by major inversions or other internal rearrangements, though we were not able to examine the repetitive terminal regions of chromosomes or the fusion junctions themselves. This collinearity, while predicted by the head-tail fusion model of Hsu et al.^4^, cannot be assessed with chromosome painting methods and would require more laborious sequence-specific probes like BACs^13,15,16,25,30^. Remarkably, we also observed that a fission event on the cervid stem (i.e., on the cervid lineage after its divergence from cattle) was reversed ~10 million years later in the *M. muntjak* lineage by a fusion of these two cervid chromosomes, regenerating the same orientation that they had in the bovid-cervid ancestor. We showed that such a fission-fusion reversal is unlikely by chance in a simple simulation of random fission and fusion events, suggesting that there may be some bias to the process. We could not have recognized the fusion in *M. muntjak* as a reversal of an earlier fission without including cow and red deer in our analysis, emphasizing the importance of multiple outgroups.

Finally, our analysis begins to describe the impact of extensive chromosome fusions on three-dimensional chromatin architecture, using Hi-C from cell culture. The high degree of sequence similarity between the muntjac genomes allowed us to directly compare the A/B compartments of the two species despite extensive chromosome fusions. While smaller-scale (sub-megabase) contacts appear to be conserved, we found that the A/B compartments showed a surprising amount of restructuring despite only ~5 million years divergence. This could be a bona fide response to massive levels of chromosome fusion, but future study of fresh samples will be needed to confirm that it is not an artifact of cell culture. The fact that the two muntjac species can produce healthy, albeit infertile, offspring^45^ suggests, however, that these differences have limited effects.

The driver of the increased rate of chromosome fusions in the muntjacs, particularly the *M. muntjak* lineage, is still under investigation^55^. We found a tenfold acceleration in the rate of chromosome change on the *M. muntjak* lineage relative to the mammalian average and twofold and threefold enhancements on the muntjac stem and in the *M. reevesi* lineage, respectively. Other muntjac species that more recently diverged from the *M. muntjak* branch have unique rearrangements^31,32^, suggesting that the fusions on this lineage did not occur all at once as a single catastrophic event, as has been described in cancer^22^. To search for genic changes correlated with rapid karyotype evolution, we examined genes with accelerated rates of evolution in *M. muntjak* and identified several potential candidates involved in chromosome maintenance. Our analysis, however, could not differentiate between genic changes that increase propensity for fusion versus subsequent adaptation to low chromosome numbers, and functional studies are needed. We hope that the availability of chromosome-scale genome sequences for the Chinese and Indian muntjacs, and the comparative analyses we have provided, can contribute to the continued understanding of this fascinating system.

## Methods

### DNA extraction and sequencing

High molecular weight DNA was extracted, as previously described^56^, from fibroblast cell lines obtained from the University of Texas Southwestern Medical Center for *M. muntjak* (female)^44^ and the University of Cambridge for *M. reevesi* (male)^11^. A 10x Genomics Chromium Genome library^23^ was prepared for each species by the DNA Technologies and Expression Analysis Cores at the University of California Davis Genome Center and sequenced on the Illumina HiSeq X by Novogene Corporation. A chromatin conformation capture library was also prepared for each species using the Dovetail Genomics Hi-C library preparation kit and sequenced on the Illumina HiSeq 4000 by the Vincent J. Coates Genomics Sequencing Laboratory at the University of California Berkeley.

### Shotgun assembly

10x Genomics linked reads were assembled de novo with Supernova (v2.0.0)^23^. Putative archaeal, bacterial, viral, and vector contamination was identified and removed by querying the assemblies using BLAST+ (v2.6.0)^57^ against the respective RefSeq and UniVec databases and removing sequences with at least 95% identity, E-value less than 1E−10, and hits aligning to more than half the scaffold size or 200 bases, using custom script general_decon.sh (v1.0). Putative mitochondrial sequence was also identified and removed by querying the assemblies using BLAST+ (v2.6.0)^57^ against their respective mitochondrial assemblies (NCBI NC_004563.1^58^ and NC_004069.1^59^) and removing sequences with at least 99% identity and E-value less than 1E−10, using custom script mt_decon.sh (v1.0). The decontamination removed 71 scaffolds totaling 836 kb from the *M. muntjak* assembly and 36 scaffolds totaling 9 kb from the *M. reevesi* assembly.

### Chromosome assembly

Hi-C reads were aligned to each assembly with Juicer (commit d3ee11b)^60^. A preliminary round of Hi-C-based scaffolding was performed with 3D-DNA (commit 745779b)^61^, and residual redundancy due to split haplotypes was manually filtered through visualization of the Hi-C contact map in Juicebox (v1.9.0)^62^, removing the smaller of any pair of duplicate scaffolds. This process removed 1.04 Gb of sequence from the *M. muntjak* assembly and 25 Mb of sequence from the *M. reevesi* assembly. The remaining scaffolds were organized into chromosomes by realigning the Hi-C reads to the deduplicated assembly with Juicer (commit d3ee11b)^60^, ordering and orienting scaffolds into chromosomes with 3D-DNA (commit 745779b)^61^, and then manually correcting in Juicebox (v1.9.0)^62^ with Juicebox Assembly Tools^63^. After correction, gaps in the assembly were filled with adapter-trimmed 10x Genomics data using custom script trim_10X.py (v1.0) and Platanus (v1.2.1)^64^.

### Final assembly release and validation

Scaffolds smaller than 1 kb in the gap-filled assembly were removed with seqtk (v1.3-r106; https://github.com/lh3/seqtk), and unplaced scaffolds were numbered in order of size from largest to smallest using SeqKit (v0.7.2-dev)^65^. Chromosomes were named based on convention from prior cytogenetic analyses^3,13,66^. Due to inconsistency in the literature^16,66–68^, MMU3_X was named as such following the standard for fused chromosomes used in *Xenopus laevis*^69^. Chromosomes in both species were oriented arbitrarily.

To validate the *M. muntjak* assembly, sequenced BACs^15,16,25^ were aligned with BWA (v0.7.17-r1188)^70^, and primary alignments were checked against the corresponding FISH locations^15,16,25^, excluding unaligned BACs or those aligned to unplaced scaffolds.

### Annotation and homology analysis

Repetitive elements were identified and classified with RepeatModeler (v1.0.11)^71^ and combined for each species with ancestral Cetartiodactyla repeats from RepBase (downloaded November 8, 2018)^72^. The assemblies were then soft masked with RepeatMasker (v4.0.7)^73^. The assemblies were annotated using Gene Model Mapper (v1.5.3)^74^ and BLAST+ (v2.6.0)^57^ with the following assemblies and annotations from Ensembl release 94^75^ as input evidence: *B. taurus* (September 2011 genebuild of GCA_000003055.3)^19^, *H. sapiens* (July 2018 genebuild of GCA_000001405.27)^27^, and *O. aries* (May 2015 genebuild of GCA_000298735.1)^26^. Coding nucleotide and peptide sequences were extracted using gff3ToGenePred and genePredToProt from the UCSC Genomics Institute (binaries downloaded March 5, 2019)^76^ with custom script postGeMoMa.py (v1.0), and functional annotation was run with InterProScan (v5.34-73.0)^28^.

Pairwise gene homology of the two muntjac annotations as well as total gene homology of the two muntjac, *B. taurus* (Ensembl release 94 September 2011 genebuild of GCA_000003055.3)^19,75^, *C. elaphus* (publication genebuild of GCA_002197005.1)^20^, and *R. tarandus*^21,77^ annotations were analyzed with OrthoVenn^78^ using an E-value cutoff of 1E−5 and an inflation value of 1.5. One-to-one orthologous muntjac genes were extracted from the pairwise OrthoVenn output with custom script extractOrthoVenn.py (v1.0), and Yang-Nielsen^79^ synonymous and nonsynonymous substitution rates were calculated with the Ks calculation script (commit 78dda1e; https://github.com/tanghaibao/bio-pipeline/tree/master/synonymous_calculation) using ClustalW2 (v2.1)^80^ and PAML (v4.7)^81^. Gene gain was identified from the full gene homology OrthoVenn output, requiring that the number of *M. muntjak* genes in an OrthoVenn cluster be greater than the number of genes found in any other analyzed species. Putative gene names of the results were extracted from the BLAST+ (v2.6.0)^57^ best hit to the *H. sapiens* proteome from UniProt^82^.

### Comparative analysis

The two muntjac assemblies were aligned to each other with cactus (commit e4d0859)^83^. After removing any ambiguous sequence with seqtk (v1.3-r106; https://github.com/lh3/seqtk), the muntjac assemblies, *C. elaphus* (GCA_002197005.1)^20^, and *R. tarandus*^21,77^ were each aligned pairwise against *B. taurus* (GCA_000003055.3)^19^ with cactus (commit e4d0859)^83^. Using custom script cactus_filter.py (v1.0), all pairwise output HAL alignment files were converted into PSL format with halLiftover (commit f7287c8)^84^. Using tools from the UCSC Genomics Institute (binaries downloaded March 5, 2019)^76^ unless noted otherwise, the PSL files were filtered and converted with pslMap, axtChain, chainPreNet, chainCleaner (commit aacca59)^85^, chainNet, netSyntenic, netToAxt, axtSort, and axtToMaf. Runs of collinearity were extracted from each pairwise MAF file by linking together local alignment blocks where the locations of species one and species two, correspondingly, were in the same orientation and were neighboring in their respective genomes without intervening aligned sequence from elsewhere in the genomes. The pairwise MAF files from the alignments against *B. taurus* were also merged with ROAST/MULTIZ (v012109)^86^, using the phylogenetic topology extracted with Newick utilities (v1.6)^87^ from a consensus tree of the species from 10kTrees^88^, and sorted with last (v912)^89^.

### Phylogeny

From the one-to-one orthologous genes of all five species identified by OrthoVenn, codons with potential fourfold degeneracy were extracted from the *B. taurus* Ensembl release 94 September 2011 genebuild^19,75^, excluding codons spanning introns, using custom script 4Dextract.py (v1.0). Using the ROAST-merged MAF file with *B. taurus* as reference, the corresponding codons were identified in the other four species, checking for amino acid conservation and excluding any codons that span two alignment blocks in the MAF file. The output FASTA file containing fourfold degenerate bases was converted into PHYLIP format with BeforePhylo (commit 0885849; https://github.com/qiyunzhu/BeforePhylo) and then analyzed with RAxML (v8.2.11)^90^ using the GTR + Gamma model of substitution with outgroup *B. taurus*.

### Estimated divergence times

We estimated divergence times from the fourfold synonymous site alignment with MEGA7 (v7.0.26)^91^, as previously described^92^. The MEGA7 time tree was constructed using the Reltime method^93^ with the GTR + Gamma model of substitution. The confidence intervals provided by TimeTree (retrieved on December 15, 2019)^37^ for all nodes except the bovid-cervid node were used as input to MEGA7. These input ranges and output times were noted in Supplementary Table 8. Confidence intervals output by MEGA7 were the same as the input confidence intervals; however, no confidence interval was output for the bovid-cervid node.

### Chromosome evolution

Pairwise alignments were extracted from the ROAST-merged MAF file using custom script extract2speciesmaf.py (v1.0) and converted into runs of collinearity following the process used in cactus_filter.py (v1.0). The runs of collinearity were visualized with Circos (v0.69-6)^94^ and, following file conversion with custom scripts mcscan_convert_links.py (v1.0) and mcscan_invert_chr.py (v1.0), with jcvi.graphics.karyotype (v0.8.12; https://github.com/tanghaibao/jcvi). Based on these visualizations and the analyzed phylogeny, with the assumption of the parsimony principle, we extracted chromosome changes using the following logic: changes that were shared in the same order and orientation between two sister species were present in the common ancestor. Any changes that did not meet this criterion were classified as lineage-specific changes. The lack of fissions or fusions on the *R. tarandus*-specific branch as well as the timings of the cervid-specific and *B. taurus*-specific fissions were derived from literature^30^.

### Chromatin conformation analysis

Hi-C reads from both species were aligned to the *M. muntjak* assembly with Juicer (commit d3ee11b)^60^, and KR normalized intra-chromosome Hi-C contact matrices were extracted with Juicer Tools (commit d3ee11b)^60^ at 1 Mb resolution. A sliding window-based localized principal component analysis (PCA) was used to call A/B compartment structure with custom script call-compartments.R (https://bitbucket.org/bredeson/artisanal). Localization of PCA1 along the diagonal of the Pearson correlation matrix (40 windows of 1 Mb each with a step size of 20) amplified the compartment signal and mitigated confounding signal from large-scale, intra-chromosome and inter-arm contacts.

Hi-C contacts from the Juicer (commit d3ee11b)^60^ merged_nodups.txt output file were split into 1 Mb and 100 kb bins using custom scripts HiCbins_1Mb.py (v1.0) and HiCbins_100kb.py (v1.0), respectively. Intra-bin and inter-bin Hi-C contacts were extracted and normalized based on the average number of contacts per bin for each species.

### Copy number analysis

To explore the three regions with variation in chromatin contacts, adapter-trimmed 10x Genomics linked reads for each species were aligned to the *M. muntjak* assembly with BWA (v0.7.17-r1188)^70^. Alignment depth was extracted with SAMtools (v1.6)^95^, and copy number was calculated from the average alignment depth per 1 Mb bin for each species.

### Reporting summary

Further information on research design is available in the Nature Research Reporting Summary linked to this article.

## Supplementary information

Description of Additional Supplementary Files

Supplementary Information

Supplementary Data 1

Reporting Summary

## Data Availability

The assemblies, annotations, and raw data for *M. muntjak* and *M. reevesi* were deposited at NCBI under BioProjects PRJNA542135 and PRJNA542137, respectively. Supporting files for the muntjac annotation and analysis are available at 10.6078/D1KT16.

## Code availability

Unless otherwise stated, custom code used in this study is available at https://github.com/abmudd/Assembly.
