# Supplementary material for: Analysis of muntjac deer genome and chromatin architecture reveals rapid karyotype evolution
Source: Commun Biol. 2020 Sep 1;3:480. doi: 10.1038/s42003-020-1096-9 (PMC7463020; doi:10.1038/s42003-020-1096-9)
Supplement: Supplementary file 1 — Description of Additional Supplementary Files [file 42003_2020_1096_MOESM1_ESM.pdf]

Description of Additional Supplementary Files

File Name: **Supplementary Data 1**
Description: Candidate positively selected genes. List of one-to-one gene orthologs between the two muntjac species with Yang-Nielsen[17] dN/dS values of 1.5 or more. Putative gene names were extracted from the BLAST+ (v2.6.0)[18] best hit to the *H. sapiens* proteome from UniProt[19]. For cases where the best hit disagreed between *M. muntjak* and *M. reevesi*, both hits were noted.
