# Supplementary material for: Analysis of muntjac deer genome and chromatin architecture reveals rapid karyotype evolution
Source: Commun Biol. 2020 Sep 1;3:480. doi: 10.1038/s42003-020-1096-9 (PMC7463020; doi:10.1038/s42003-020-1096-9)
Supplement: Supplementary file 2 — Supplementary Information [file 42003_2020_1096_MOESM2_ESM.pdf]

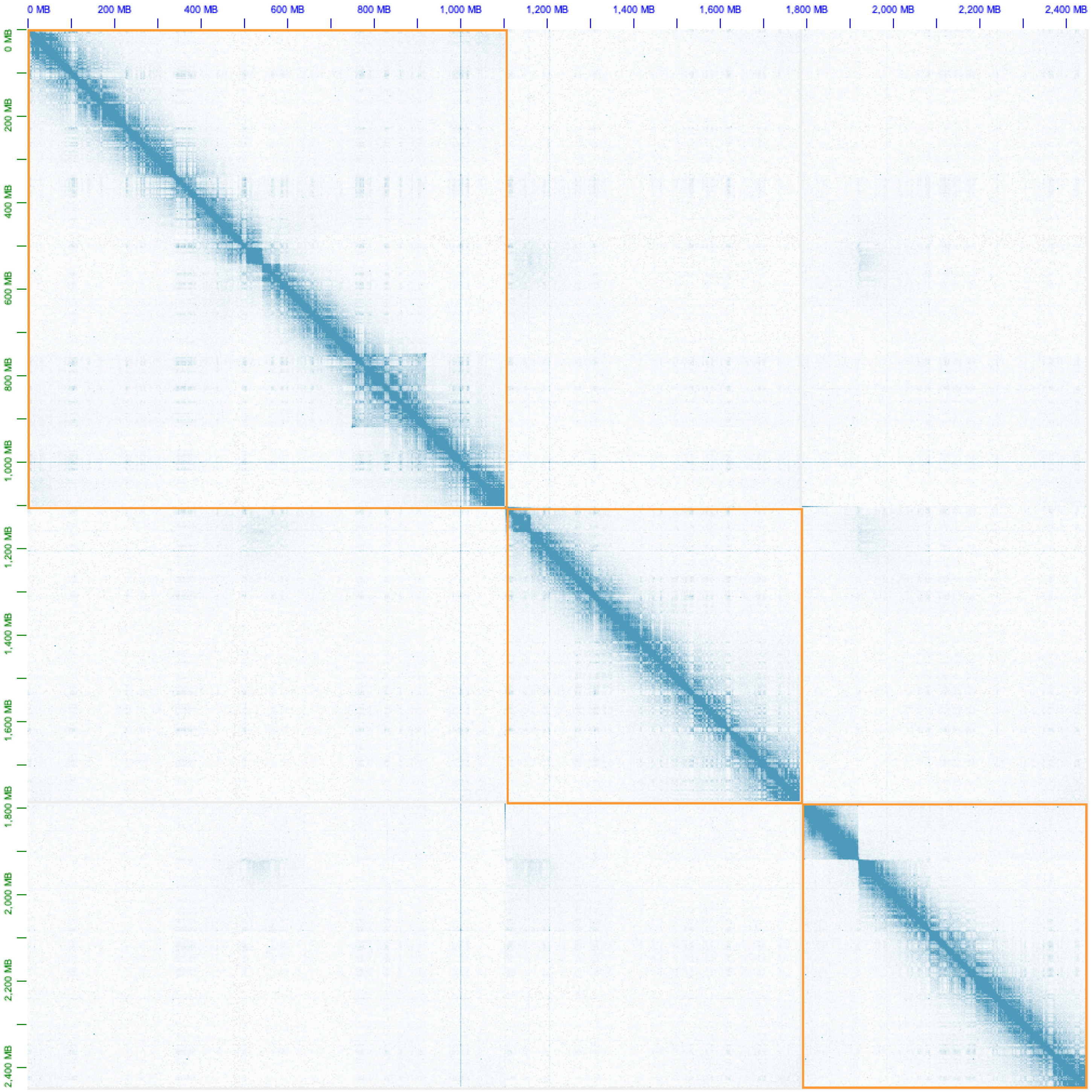

Supplementary Figure 1. Genome-wide Hi-C contact map of *M. muntjak*. *M. muntjak* Hi-C data mapped to the *M. muntjak* assembly (*x* and *y* axes). The three *M. muntjak* chromosomes are outlined in orange and are shown in ascending order (from upper-left to lower-right). The intensity of blue pixels is proportional to the contact frequency between *x-y* pairs of genomic loci. The highest intensity pixels are along the diagonal of each chromosome, indicating a high degree of contacts between loci in close proximity. The checkerboard/striped patterns within chromosomes reflect reduced contact frequency between neighboring loci and increased contacts between more distant loci due to the three-dimensional chromatin folding (i.e., A/B compartment) structure within the nucleus.

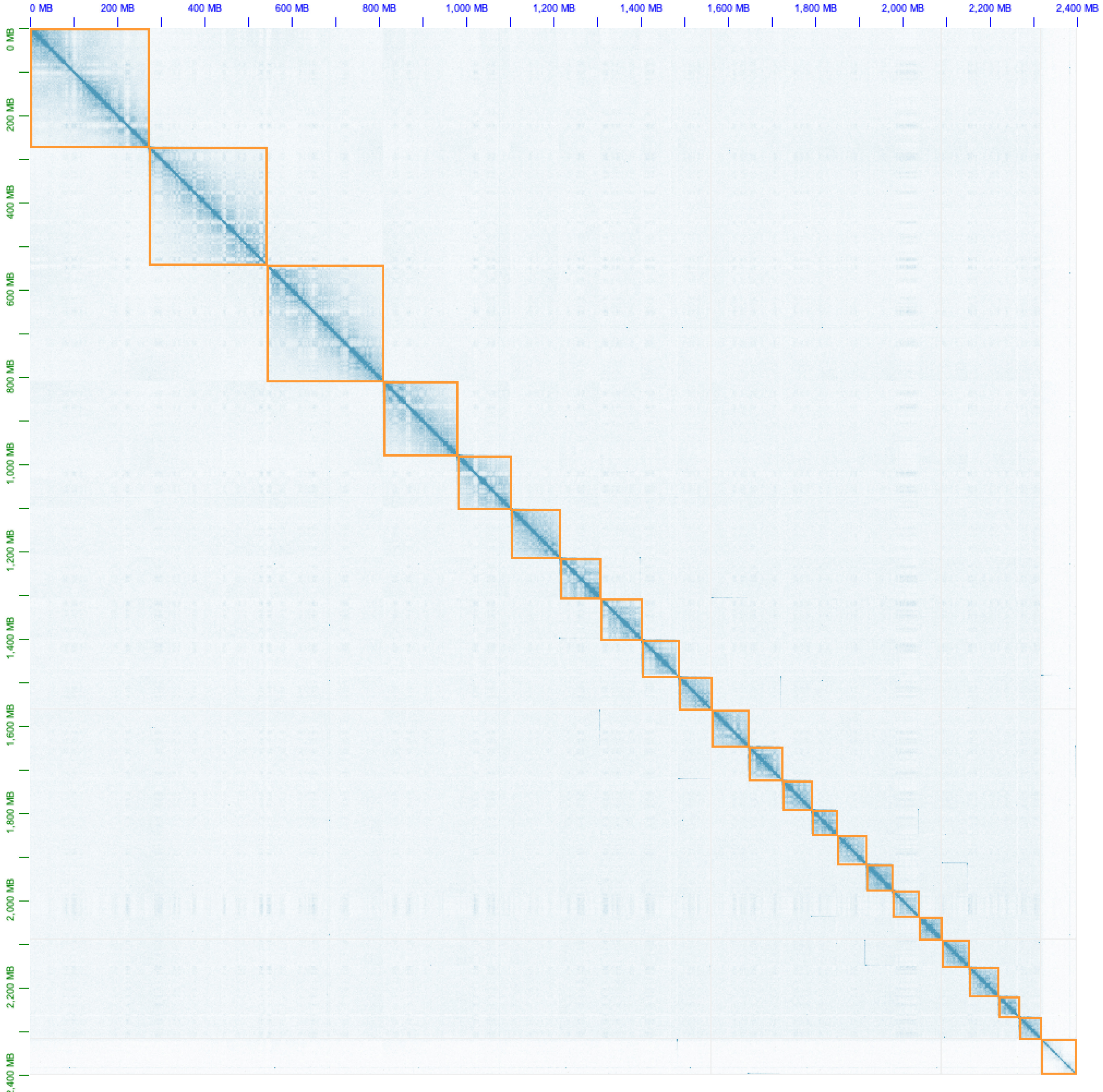

Supplementary Figure 2. Genome-wide Hi-C contact map of *M. reevesi*. *M. reevesi* Hi-C data mapped to the *M. reevesi* assembly (*x* and *y* axes). The 23 *M. reevesi* chromosomes are outlined in orange and are shown in ascending order (from upper-left to lower-right). The intensity of blue pixels is proportional to the contact frequency between *x*-*y* pairs of genomic loci. The highest intensity pixels are along the diagonal of each chromosome, indicating a high degree of contacts between loci in close proximity. The checkerboard/striped patterns within chromosomes reflect reduced contact frequency between neighboring loci and increased contacts between more distant loci due to the three-dimensional chromatin folding (i.e., A/B compartment) structure within the nucleus.

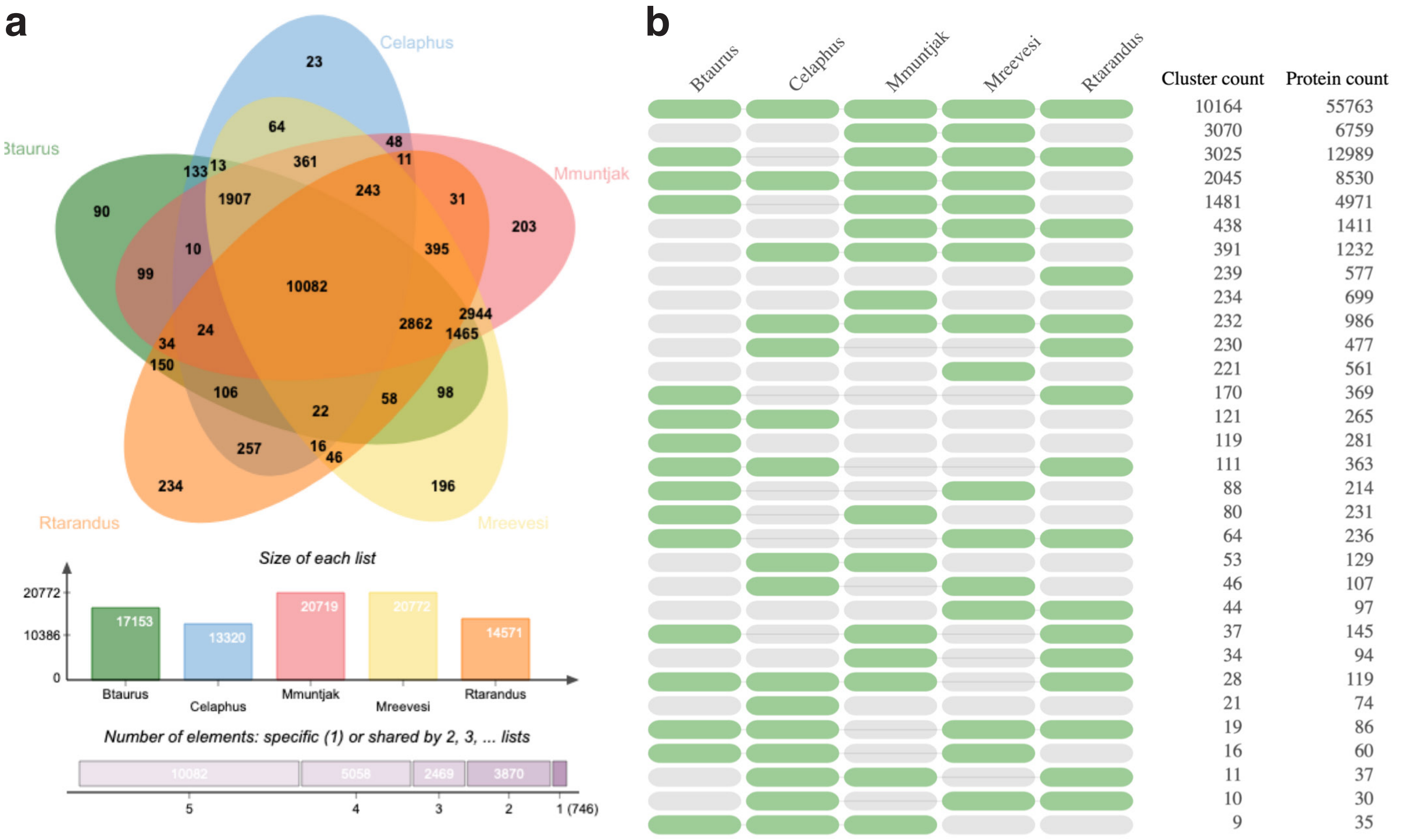

Supplementary Figure 3. Quantification of orthologous protein-coding genes in four cervid species and cow. (a) Venn diagram of gene homology between the two muntjac annotations, *B. taurus* (Ensembl release 94 September 2011 genebuild of GCA_000003055.3)[1,2], *C. elaphus* (publication genebuild of GCA_002197005.1)[3], and *R. tarandus*[4,5] annotations analyzed with OrthoVenn[6] and (b) the occurrence table of gene homology clusters between these species reanalyzed with OrthoVenn2[7] for visualization purposes. In the occurrence table, the green and grey ovals represent the presence or absence, respectively, of that species in the OrthoVenn2 clustering. The number of clusters and proteins were provided for all species combinations.

a

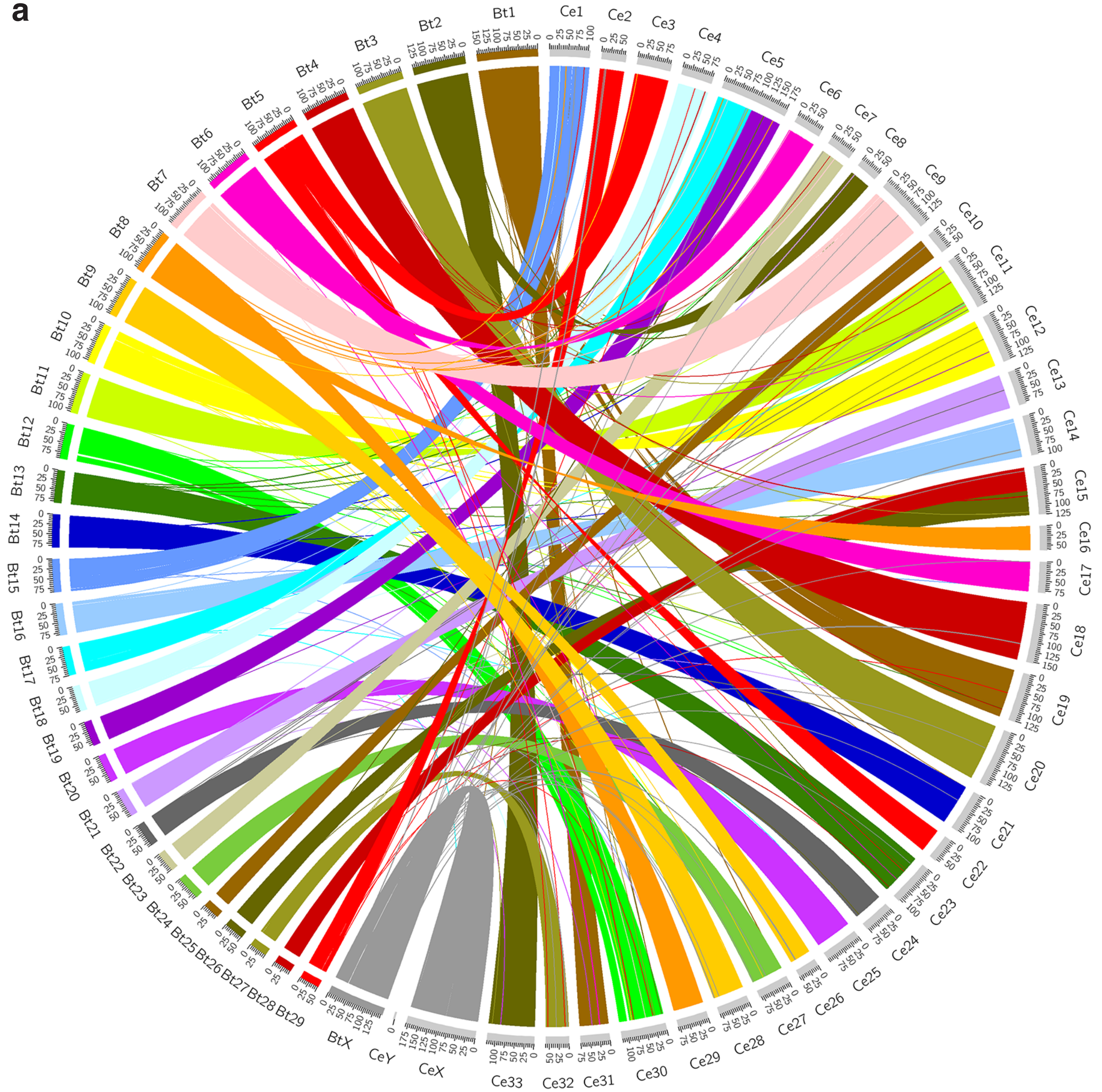
Bt1
Bt2
Bt3
Bt4
Bt5
Bt6
Bt7
Bt8
Bt9
Bt10
Bt11
Bt12
Bt13
Bt14
Bt15
Bt16
Bt17
Bt18
Bt19
Bt20
Bt21
Bt22
Bt23
Bt24
Bt25
Bt26
Bt27
Bt28
Bt29
BtX
CeY
CeX
Ce33
Ce32
Ce31
Ce30
Ce29
Ce28
Ce27
Ce26
Ce25
Ce24
Ce23
Ce22
Ce21
Ce20
Ce19
Ce18
Ce17
Ce16
Ce15
Ce14
Ce13
Ce12
Ce11
Ce10
Ce9
Ce8
Ce7
Ce6
Ce5
Ce4
Ce3
Ce2
Ce1

b

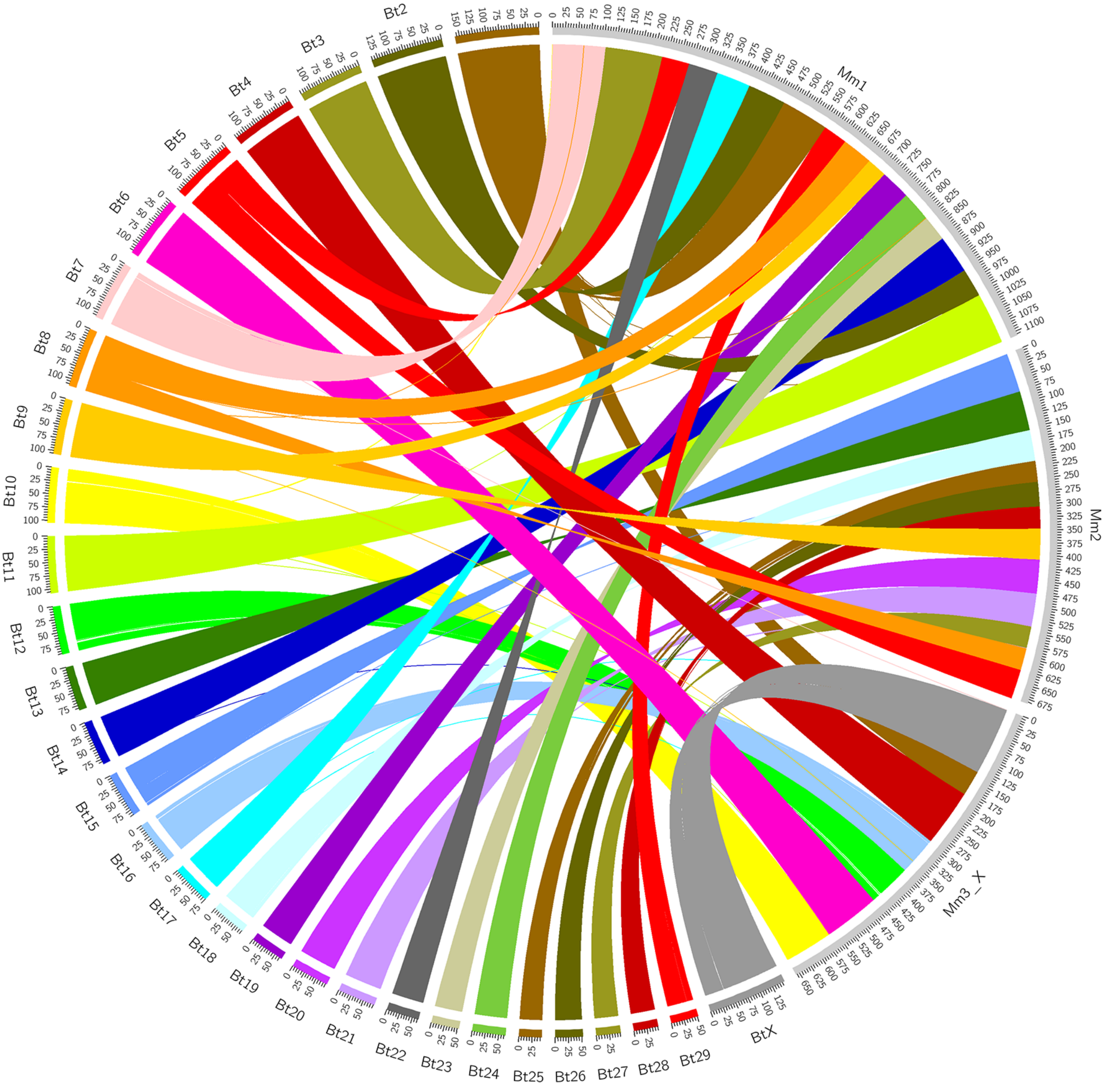

Bt1
Bt2
Bt3
Bt4
Bt5
Bt6
Bt7
Bt8
Bt9
Bt10
Bt11
Bt12
Bt13
Bt14
Bt15
Bt16
Bt17
Bt18
Bt19
Bt20
Bt21
Bt22
Bt23
Bt24
Bt25
Bt26
Bt27
Bt28
Bt29
BtX
Mm1
Mm2
Mm3_X

c

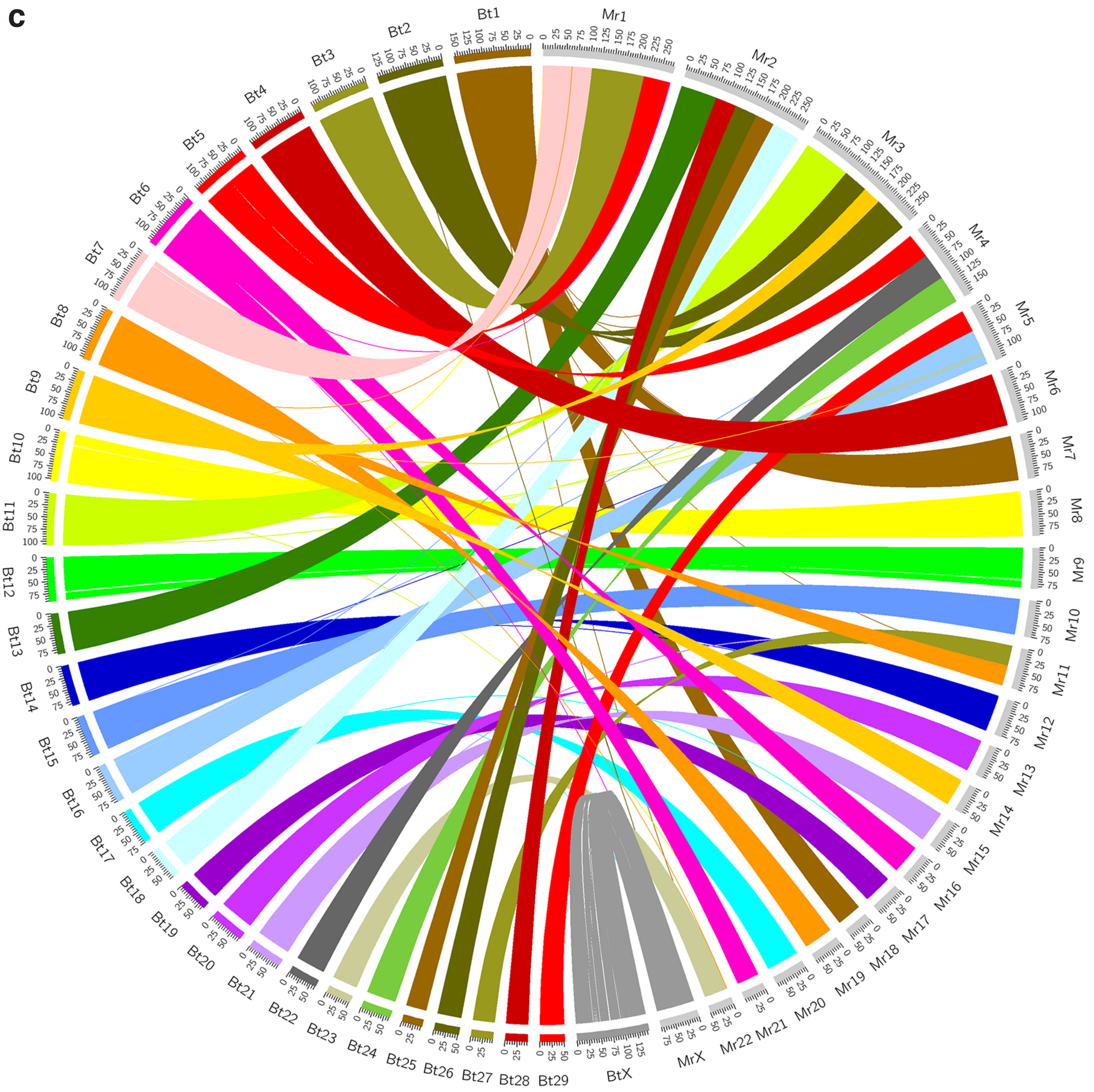
Bt1
Bt2
Bt3
Bt4
Bt5
Bt6
Bt7
Bt8
Bt9
Bt10
Bt11
Bt12
Bt13
Bt14
Bt15
Bt16
Bt17
Bt18
Bt19
Bt20
Bt21
Bt22
Bt23
Bt24
Bt25
Bt26
Bt27
Bt28
Bt29
BtX
Mr1
Mr2
Mr3
Mr4
Mr5
Mr6
Mr7
Mr8
Mr9
Mr10
Mr11
Mr12
Mr13
Mr14
Mr15
Mr16
Mr17
Mr18
Mr19
Mr20
Mr21
Mr22
MrX

d

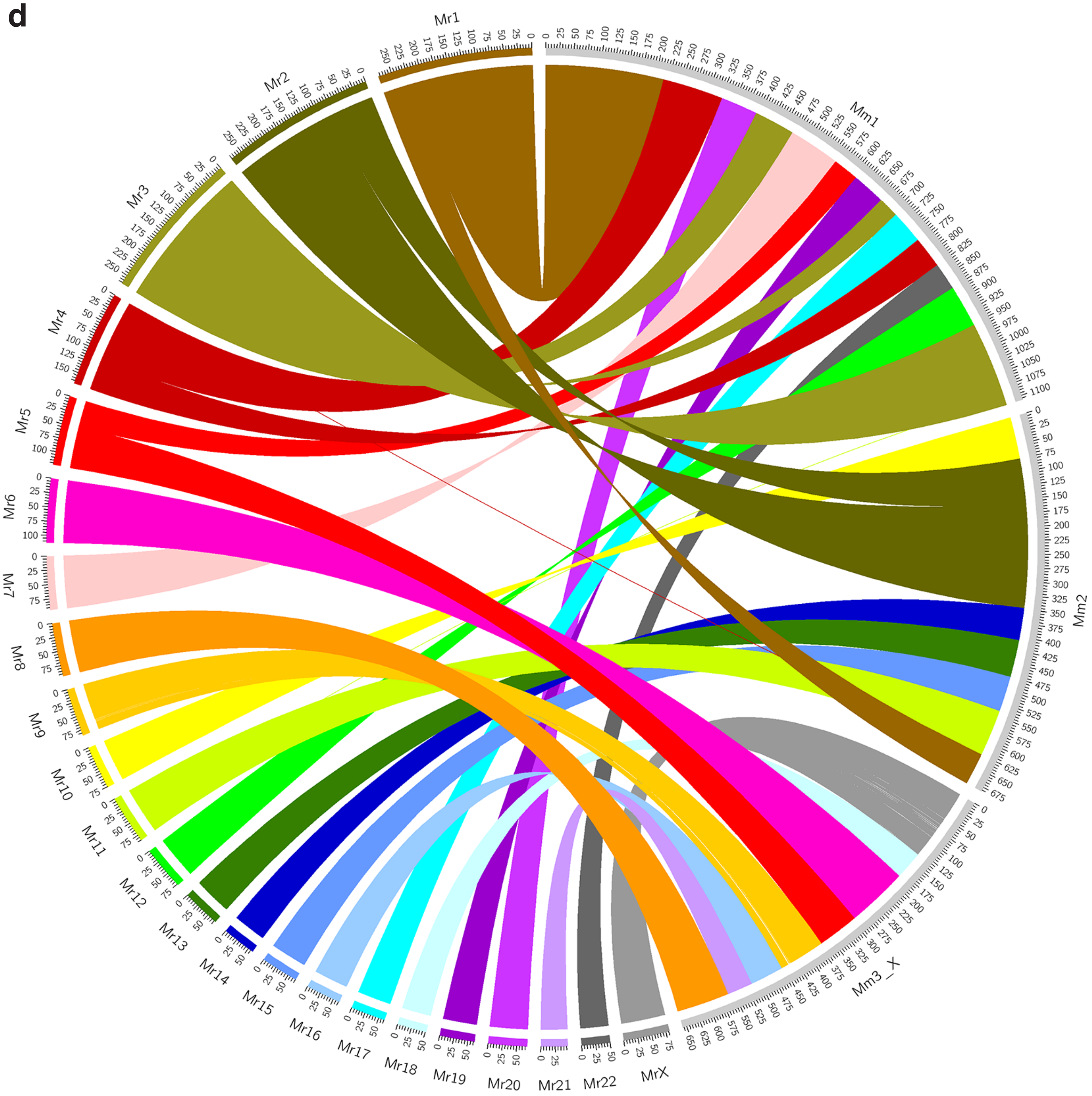
Mr1
Mr2
Mr3
Mr4
Mr5
Mr6
Mr7
Mr8
Mr9
Mr10
Mr11
Mr12
Mr13
Mr14
Mr15
Mr16
Mr17
Mr18
Mr19
Mr20
Mr21
Mr22
MrX
Mm1
Mm2
Mm3_X

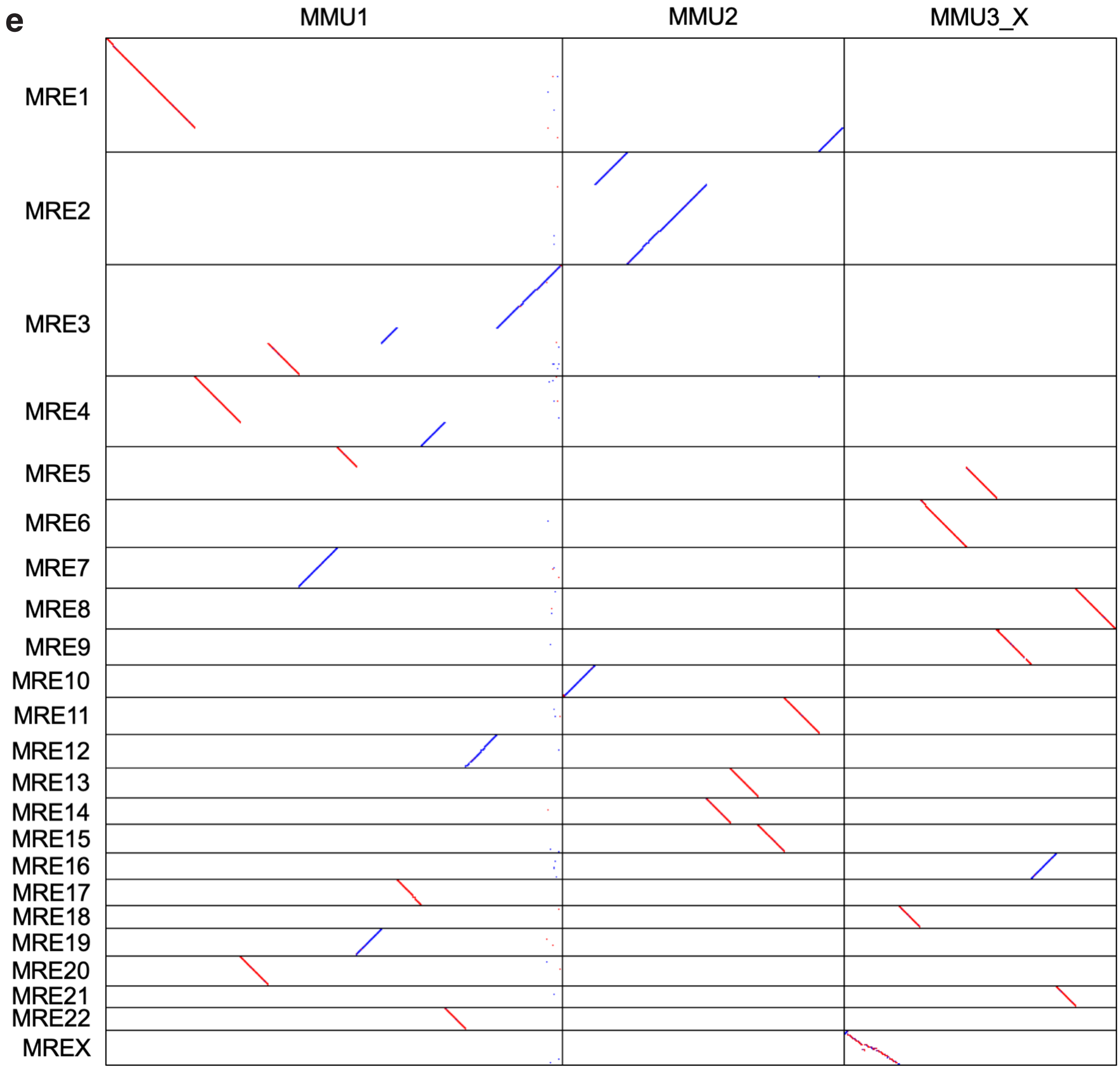

Supplementary Figure 4. Pair-wise comparisons between cow and cervid chromosome-scale assemblies. a–d Circos (v0.69-6)[8] plots with runs of collinearity containing at least 25 kb of aligned sequence between **a** *B. taurus* (left, Bt) and *C. elaphus* (right, Ce), **b** *B. taurus* (left, Bt) and *M. muntjak* (right, Mm), **c** *B. taurus* (left, Bt) and *M. reevesi* (right, Mr), and **d** *M. reevesi* (left, Mr) and *M. muntjak* (right, Mm). **e** Dot plot of runs of collinearity containing at least 25 kb of aligned sequence between *M. reevesi* (left, MRE) and *M. muntjak* (top, MMU) visualized with last (v979)[9].

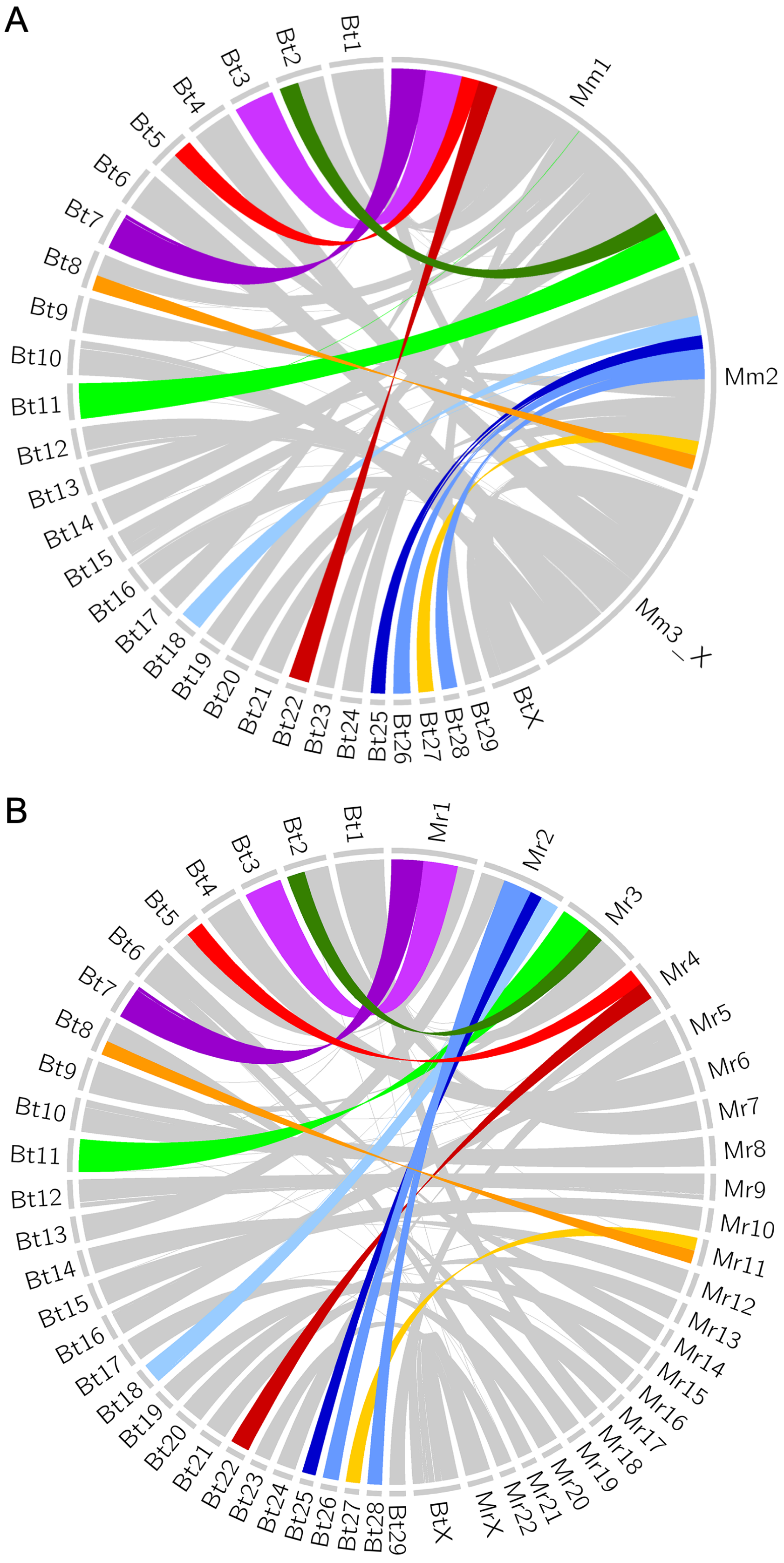

Supplementary Figure 5. Circos plots highlighting fussions shared by *M. muntjak* and *M. reevesi*. Circos (v0.69-6)[8] plots using runs of collinearity containing at least 25 kb of aligned sequence between **a** *B. taurus* (left, Bt) and *M. muntjak* (right, Mm) and **b** B. taurus (left, Bt) and *M. reevesi* (right, Mr) specifying the six shared muntjac fusions: BTA7/BTA3 (purple), BTA5prox/BTA22 (red), BTA2dist/BTA11 (green), BTA18/BTA25/BTA26_28 (blue), and BTA27/BTA8dist (orange).

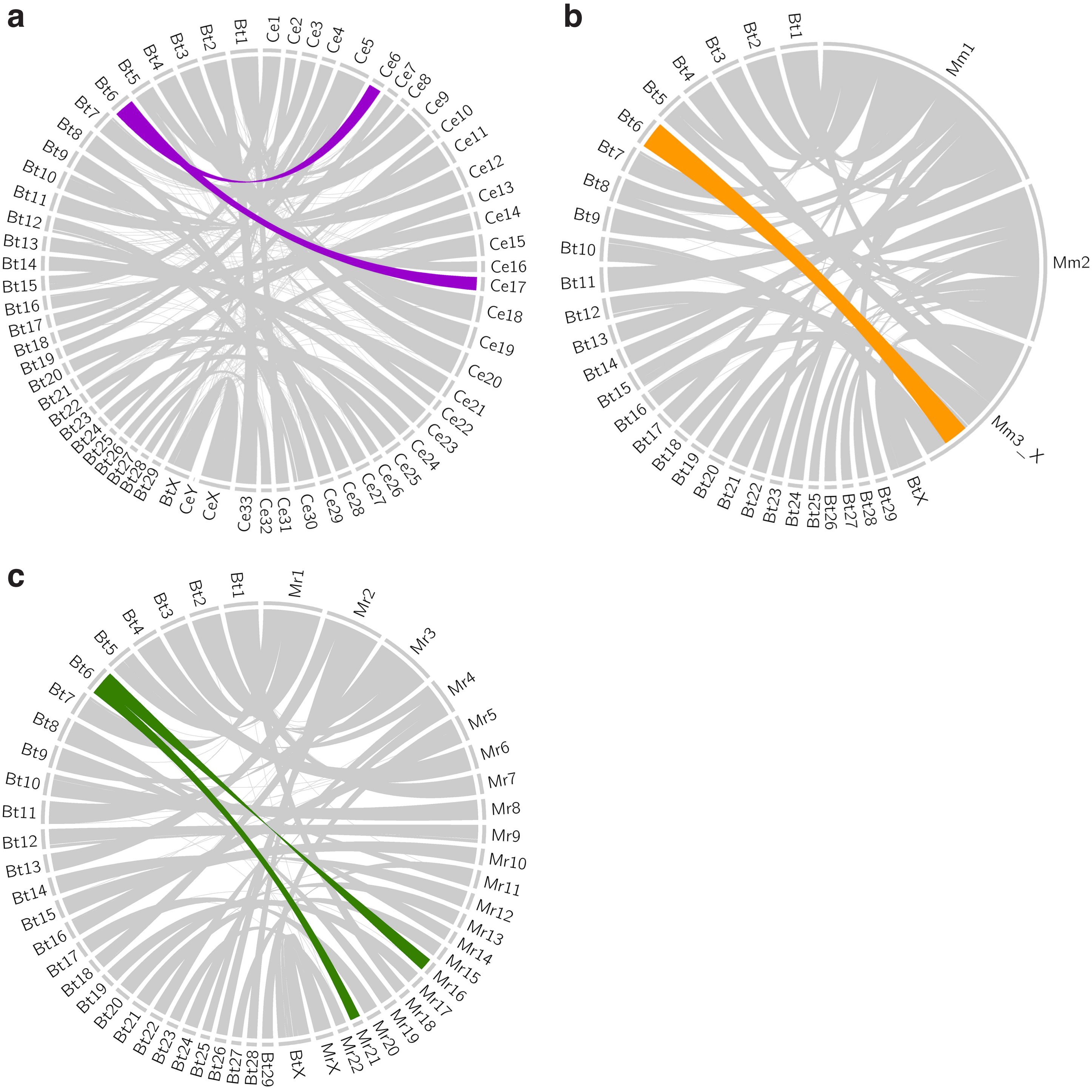

Supplementary Figure 6. Circos plots highlighing fissions in the cervid lineage corresponding to cow chromsome six. Circos (v0.69-6)[8] plots using runs of collinearity containing at least 25 kb of aligned sequence between **a** *B. taurus* (left, Bt) and *C. elaphus* (right, Ce) with the fission of BTA6 in purple; **b** *B. taurus* (left, Bt) and *M. muntjak* (right, Mm) with the fission-fusion reversal of BTA6 in orange; and **c** *B. taurus* (left, Bt) and *M. reevesi* (right, Mr) with the fission of BTA6 in green.

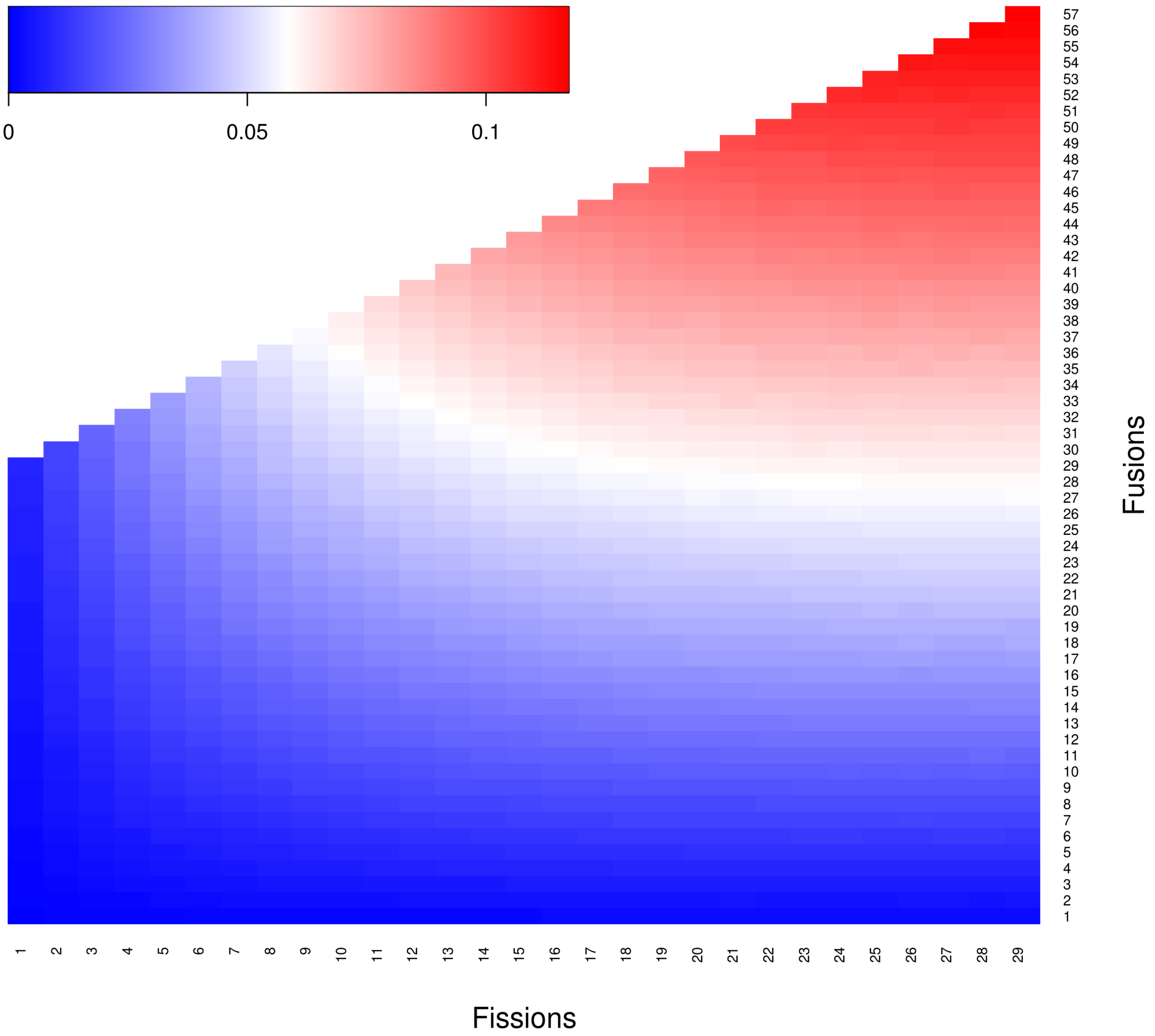

Supplementary Figure 7. Chromosome fission-fusion reversal simulation. Heatmap of probabilities where at least one fusion reverses a prior fission modeled to 1 million iterations for each possible scenario from a starting karyotype of n=29, using custom script run_fission_fusion.sh (v1.0).

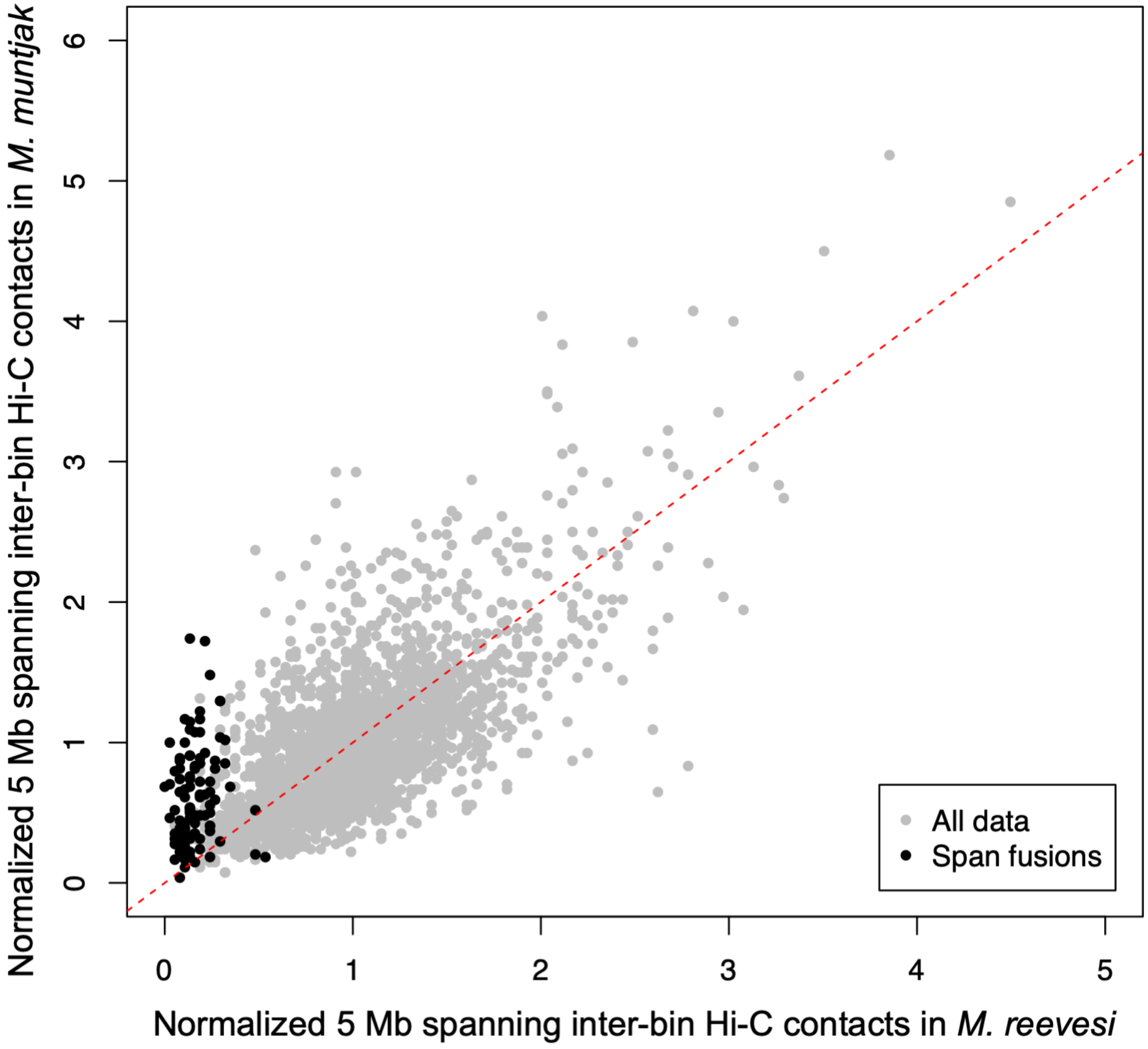

Supplementary Figure 8. Chromatin contacts boundaries not maintained at fusion sites after chromosome fusion events. Normalized 1 Mb inter-bin Hi-C contacts between bins 5 Mb apart for *M. muntjak* (*y* axis) vs. *M. reevesi* (*x* axis) with the inter-bin contacts that span across, but do not include, the *M. muntjak* lineage-specific fusion sites (see **Supplementary Table 7**) colored black. The expected result of conserved Hi-C contacts is represented with a dashed red line.

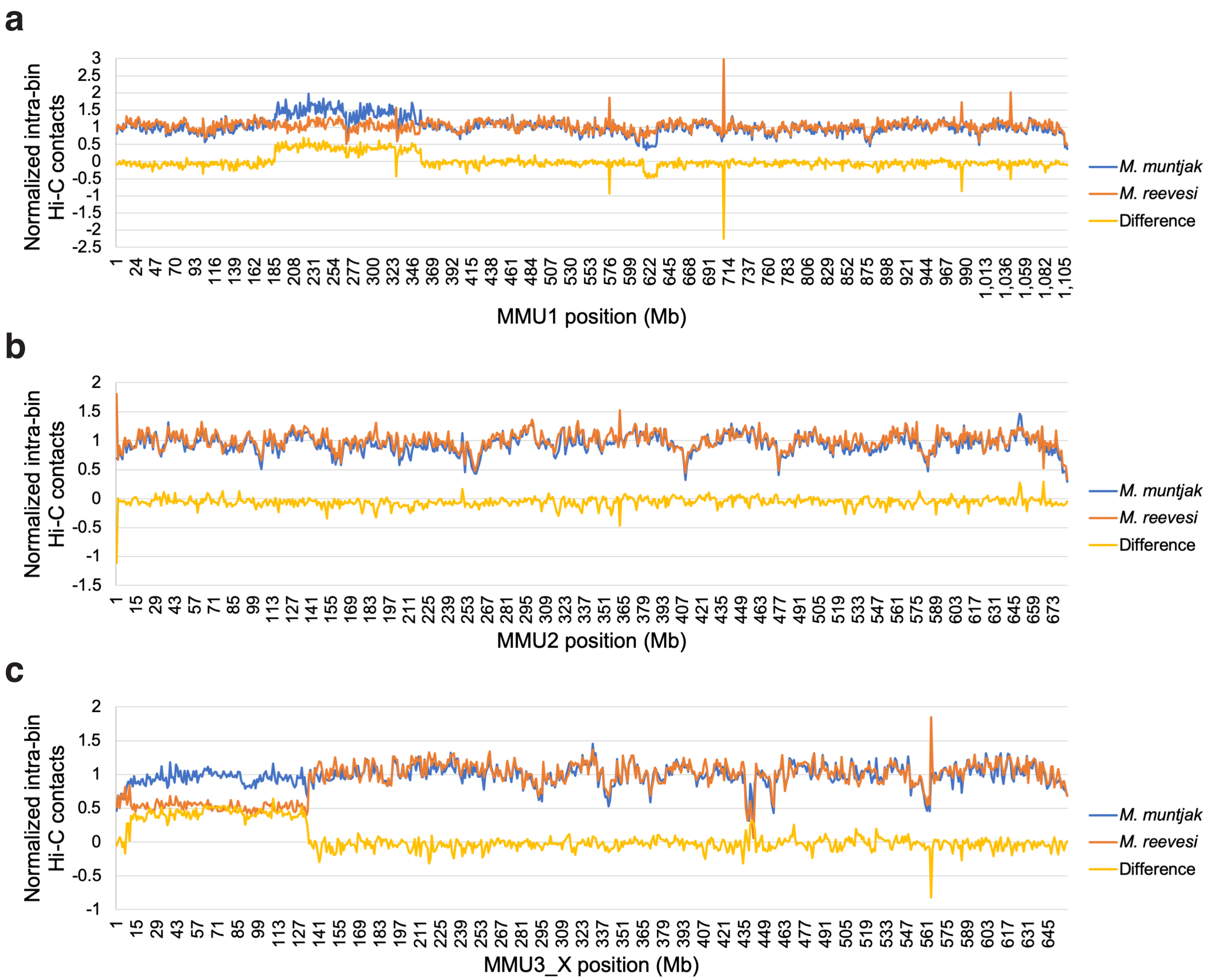

Supplementary Figure 9. Copy number differences between *M. muntjak* and *M. reevesi*. Using a bin size of 1 Mb and the *M. muntjak* assembly as the reference, normalized intra-bin Hi-C contacts for *M. muntjak* (blue) and *M. reevesi* (orange) at each position on **a** MMU1, **b** MMU2, and **c** MMU3_X. The difference of *M. muntjak* contacts minus *M. reevesi* contacts is displayed in yellow.

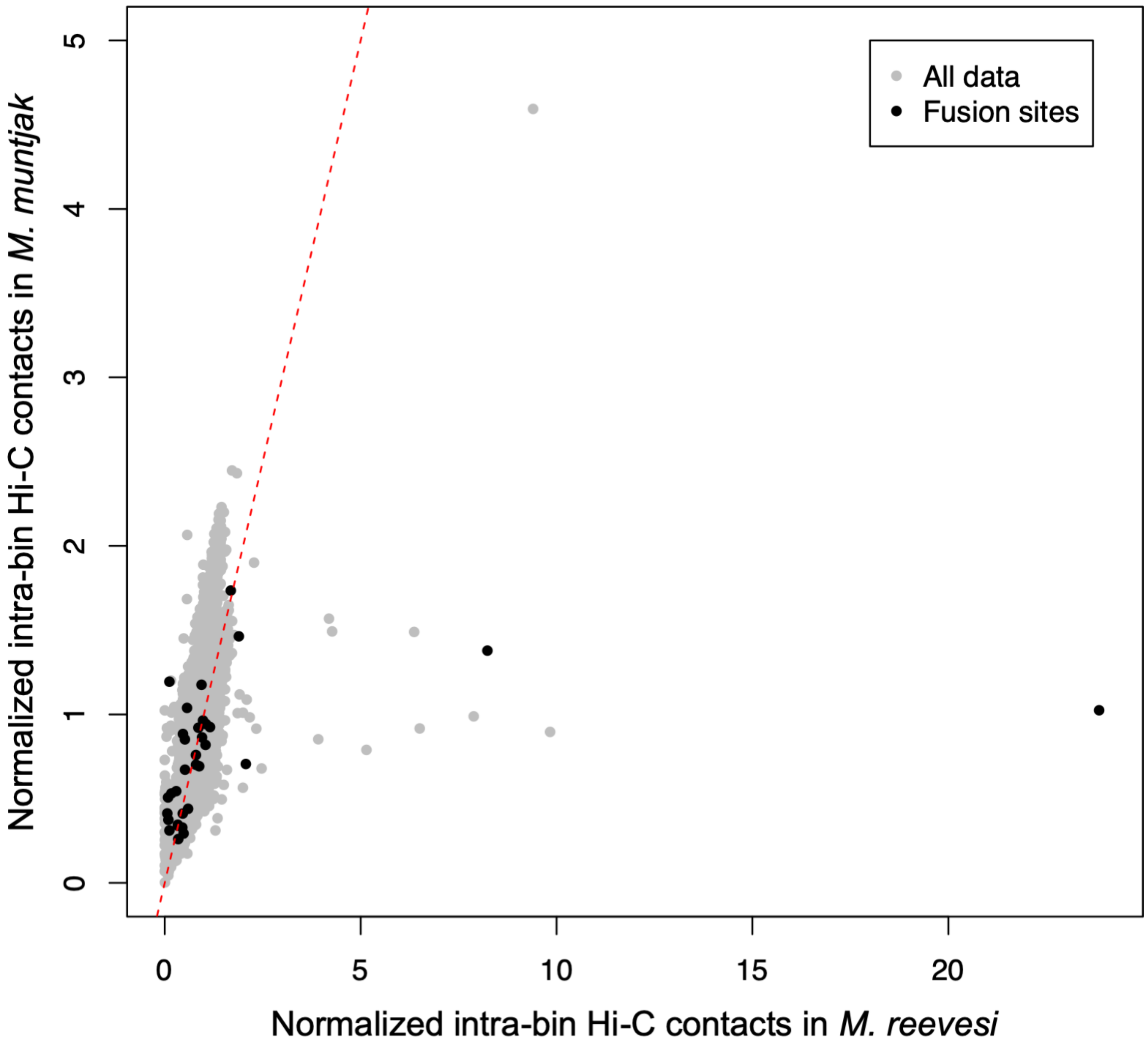

Supplementary Figure 10. Chromatin contacts conserved within syntenic boundaries after chromosome fusion events. Normalized 100 kb intra-bin Hi-C contacts for *M. muntjak* (*y* axis) vs. *M. reevesi* (*x* axis) with bins containing the *M. muntjak* lineage-specific fusion sites (see **Table S7**) colored black. The expected result of conserved Hi-C contacts is represented with a dashed red line. For fusion site ranges spanning two bins, the bin containing the majority of the fusion site range was deemed to be the fusion site bin. For fusion site ranges spanning three or more bins, the middle 100 kb bin(s) was deemed to be the fusion site bin(s).

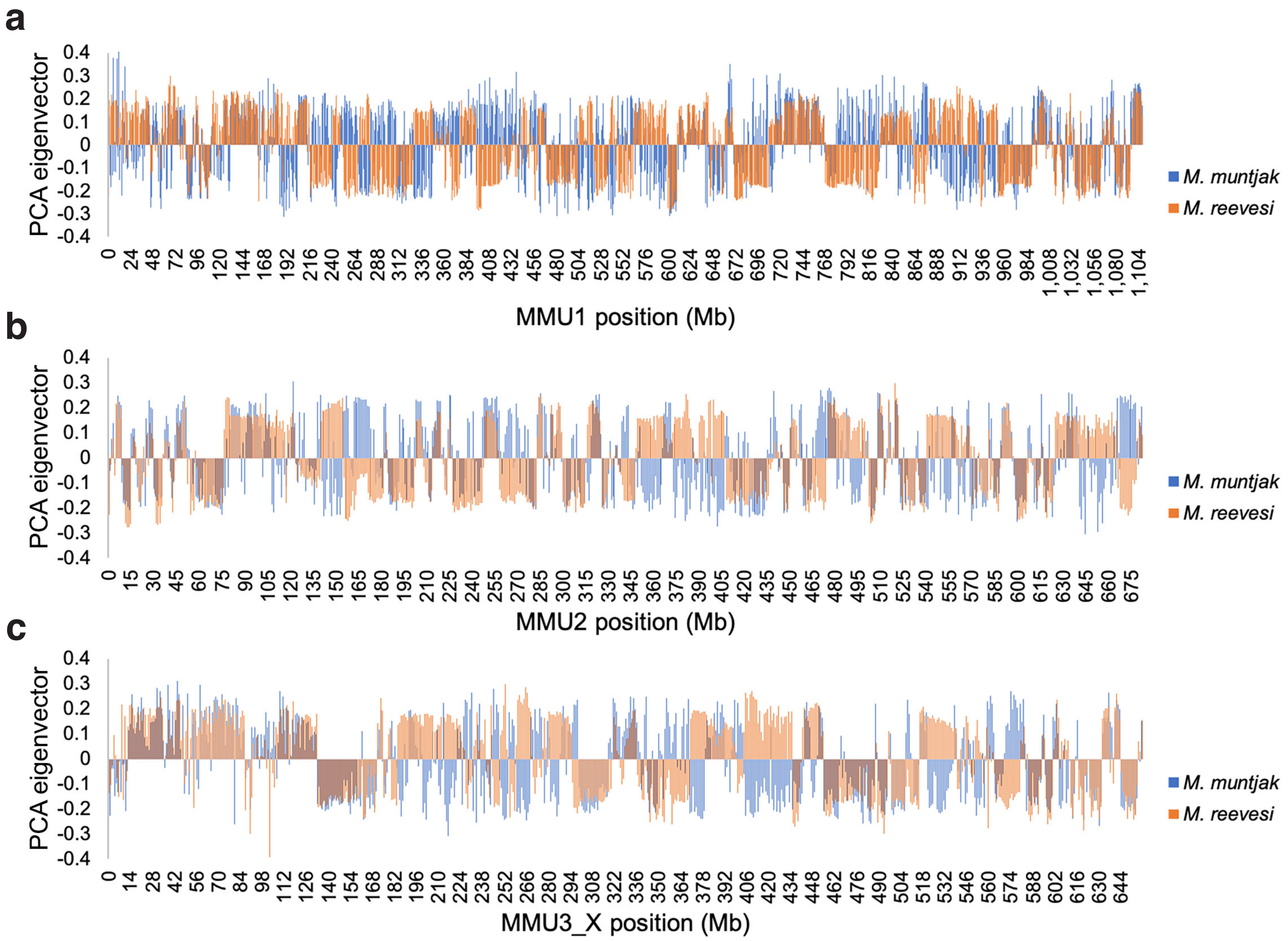

Supplementary Figure 11. Chromatin A/B compartment conservation and change. Identification of A/B compartment boundaries for *M. muntjak* (blue) and *M. reevesi* (orange) on the *M. muntjak* assembly using localized principal component analysis (PCA) eigenvectors calculated by call-compartments.R (https://bitbucket.org/bredeson/artisanal). **a** MMU1, **b** MMU2, **c** MMU3_X.

Supplementary Table 1. Summary of the muntjac genome assemblies. Statistics were calculated using assembly-stats (commit 506a640; https://github.com/sanger-pathogens/assembly-stats).

Genomic feature	*M. muntjak*	*M. reevesi*
Total scaffold length, bp	2,573,529,099	2,579,575,442
Number of scaffolds	25,651	29,705
Scaffold N50 length, bp	682,452,208	94,101,870
Total contig length, bp	2,518,738,577	2,514,747,046
Number of contigs	49,270	53,090
Contig N50 length, bp	215,534	225,142
Contigs sequence in chromosomes, %	95.06	92.93
Contig GC content, %	41.59	41.59
Masked contig repeat sequence, %	40.33	40.06
Number of genes	25,737	26,044
Genes with functional annotation, %	98.11	98.15
Average number of exons per gene	7.83	7.77
Median predicted peptide length, aa	328	326
Median exon size, bp	124	124
Median intron size, bp	921	911

Supplementary Table 2. DNA sequencing generated for genome assembly. Excludes index reads.

Species	Library type	Total number of reads	Total bases sequenced
M. muntjak NCBI BioProject PRJNA542135	10x Genomics Chromium Genome	768,921,264	115,338,189,600
	Dovetail Genomics Hi-C	521,749,568	78,784,184,768
M. reevesi NCBI BioProject PRJNA542137	10x Genomics Chromium Genome	696,864,964	104,529,744,600
	Dovetail Genomics Hi-C	530,002,086	80,030,314,986

Supplementary Table 3. Pairwise nucleotide divergence in substitutions per site at fourfold degenerate sites. Extracted from the RAxML (v8.2.11)[10] phylogenetic tree for the examined species using Newick utilities (v1.6)[11].

Species	*B. taurus*	*C. elaphus*	*M. muntjak*	*M. reevesi*
C. elaphus	0.0550	–	–	–
M. muntjak	0.0606	0.0267	–	–
M. reevesi	0.0599	0.0259	0.0130	–
R. tarandus	0.0592	0.0298	0.0355	0.0347

Supplementary Table 4. Locations of the six cervid-specific fissions relative to the *B. taurus* genome assembly.

B. taurus **chromosome**	**Using runs of collinearity from *C. elaphus***	**Using runs of collinearity from *M. muntjak***	**Using runs of collinearity from *M. reevesi***
BTA1	58,941,477 – 58,978,602	57,645,593 – 57,778,258	57,645,547 – 57,746,127
BTA2	93,282,776 – 93,424,724	79,668,309 – 79,719,766	79,668,935 – 79,719,765
BTA5	70,623,938 – 70,699,763	57,880,818 – 58,822,584	57,880,818 – 60,196,482
BTA6	63,301,740 – 63,370,450	Fusion reversal	68,435,554 – 68,455,313
BTA8	64,071,291 – 64,114,095	67,266,180 – 67,499,444	67,369,805 – 67,497,566
BTA9	63,670,677 – 64,013,115	64,824,832 – 65,087,945	64,824,832 – 65,087,945

Supplementary Table 5. Muntjac chromosome fusions. Locations of the shared fusion events in the *M. muntjak* and *M. reevesi* genome assemblies using runs of collinearity from *B. taurus*.

B. taurus **fused chromosomes**	***M. muntjak*** **region**	***M. reevesi*** **region**
BTA7/BTA3	MMU1: 103,142,901 – 103,201,151	MRE1: 103,650,999 – 103,852,521
BTA5prox/BTA22	MMU1: 267,859,762 – 267,926,350	MRE4: 52,627,734 – 52,781,577
BTA2dist/BTA11	MMU1: 1,006,153,244 – 1,006,638,886	MRE3: 100,711,306 – 101,302,993
BTA18/BTA25	MMU2: 216,351,804 – 216,390,051	MRE2: 210,159,178 – 210,200,937
BTA25/BTA26	MMU2: 256,956,082 – 257,281,016	MRE2: 169,793,458 – 170,063,149
BTA26_28 (*B. taurus* fission)	MMU2: 305,072,202 – 305,072,202	MRE2: 121,918,267 – 122,082,397
BTA27/BTA8dist	MMU2: 580,274,743 – 582,942,905	MRE11: 40,769,993 – 43,560,918

Supplementary Table 6. *M. reevesi* fusion junctions. Locations of the six unique fusion events in the *M. reevesi* genome assembly were derived from one-to-one orthologs between *M. muntjak* and *M. reevesi* and then refined using runs of collinearity from *B. taurus* and *M. muntjak* against *M. reevesi*.

***B. taurus* fused chromosomes**	***M. reevesi* chromosome**	***M. reevesi* start**	***M. reevesi* end**
BTA7_3/BTA5dist	MRE1	216,558,007	216,594,231
BTA18_25_26_28/BTA13	MRE2	78,441,940	78,562,328
BTA2prox/BTA9dist	MRE3	154,290,838	154,298,872
BTA9dist/BTA2dist_11	MRE3	191,988,183	192,099,419
BTA5prox_22/BTA24	MRE4	111,704,918	111,709,617
BTA29/BTA16	MRE5	47,104,391	47,224,935

Supplementary Table 7. *M. muntjak* fusion junctions. Locations of the 26 unique fusion events in the *M. muntjak* genome assembly were derived from one-to-one orthologs between *M. muntjak* and *M. reevesi* and then refined using runs of collinearity from *B. taurus* and *M. reevesi* against *M. muntjak*.

***B. taurus* fused chromosomes**	***M. muntjak* chromosome**	***M. muntjak* start**	***M. muntjak* end**
BTA7_3/BTA5prox_22	MMU1	215,667,096	215,740,550
BTA5prox_22/BTA17	MMU1	326,596,489	326,664,606
BTA17/BTA2prox	MMU1	394,376,597	394,423,120
BTA2prox/BTA1dist	MMU1	468,279,958	468,421,169
BTA1dist/BTA29	MMU1	562,054,424	562,154,407
BTA29/ BTA8prox	MMU1	609,147,303	609,442,186
BTA8prox/BTA9dist	MMU1	669,798,392	669,917,570
BTA9dist/BTA19	MMU1	707,332,274	707,411,696
BTA19/BTA24	MMU1	767,481,614	767,858,594
BTA24/BTA23	MMU1	825,563,679	825,664,460
BTA23/BTA14	MMU1	875,352,976	875,473,556
BTA14/BTA2dist_11	MMU1	952,277,739	952,439,995
BTA15/BTA13	MMU2	76,554,689	76,587,068
BTA13/BTA18_25_26_28	MMU2	155,096,035	155,468,627
BTA18_25_26_28/BTA9prox	MMU2	348,208,540	348,522,144
BTA9prox/BTA20	MMU2	407,305,863	407,476,405
BTA20/BTA21	MMU2	474,891,789	475,146,554
BTA21/BTA27_8dist	MMU2	540,052,390	540,055,842
BTA27_8dist/BTA5dist	MMU2	624,503,504	624,522,918
BTAX/BTA1prox	MMU3_X	133,000,163	133,001,250
BTA1prox/BTA4	MMU3_X	184,069,851	184,122,591
BTA4/BTA16	MMU3_X	295,103,485	295,251,858
BTA16/BTA12	MMU3_X	370,301,578	370,307,164
BTA12/BTA6prox	MMU3_X	454,989,747	454,992,643
BTA6prox/BTA6dist	MMU3_X	516,012,504	516,138,154
BTA6dist/BTA10	MMU3_X	562,995,659	563,046,092

Supplementary Table 8. Estimated divergence times within cervids and between Bovidae and Cervidae. Estimated divergence times in million years were calculated with MEGA7 (v7.0.26)[12] and compared with published sources. TimeTree data was retrieved on December 15, 2019[13]. Times from Toljagić et al.[14] and Zurano et al.[15] were derived from mitochondrial sequences, whereas times from Chen et al.[16] were from the alignment of nuclear genomes.

Node	This work	TimeTree estimated times[13]	TimeTree confidence intervals[13]	Toljagić et al.[14]	Zurano et al. (dataset1)[15]	Chen et al.[16]
M. muntjak – M. reevesi	4.9	4.7	2.9 – 6.5	4.1	4.2	3.2
Muntiacus – Cervus	11.8	13.6	8.6 – 14.4	11.4	10.9	9.1
(*Muntiacus, Cervus*) – *Rangifer*	13.6	13.6	10.6 – 16.6	14.2	13.5	10.6
Bovidae – Cervidae	22.8	27.3	23.4 – 28.7	22.7	22.0	20.3

Supplementary References

1. Zimin, A. V. et al. A whole-genome assembly of the domestic cow, *Bos taurus*. *Genome Biol.* **10**, R42 (2009).
2. Cunningham, F. et al. Ensembl 2019. *Nucleic Acids Res.* **47**, D745–D751 (2019).
3. Bana, N. Á. et al. The red deer *Cervus elaphus* genome CerEla1.0: sequencing, annotating, genes, and chromosomes. *Mol. Genet. Genomics* **293**, 665–684 (2018).
4. Li, Z. et al. Draft genome of the reindeer (*Rangifer tarandus*). *Gigascience* **6**, 1–5 (2017).
5. Li, Z. et al. Draft genomic data of the reindeer (*Rangifer tarandus*). *GigaScience Database.* https://doi.org/10.5524/100370 (2017).
6. Wang, Y., Coleman-Derr, D., Chen, G. & Gu, Y. Q. OrthoVenn: a web server for genome wide comparison and annotation of orthologous clusters across multiple species. *Nucleic Acids Res.* **43**, W78–W84 (2015).
7. Xu, L. et al. OrthoVenn2: a web server for whole-genome comparison and annotation of orthologous clusters across multiple species. *Nucleic Acids Res.* **47**, W52–W58 (2019).
8. Krzywinski, M. et al. Circos: an information aesthetic for comparative genomics. *Genome Res.* **19**, 1639–1645 (2009).
9. Kielbasa, S. M., Wan, R., Sato, K., Horton, P. & Frith, M. C. Adaptive seeds tame genomic sequence comparison. *Genome Res.* **21**, 487–493 (2011).
10. Stamatakis, A. RAxML version 8: a tool for phylogenetic analysis and post-analysis of large phylogenies. *Bioinformatics* **30**, 1312–1313 (2014).
11. Junier, T. & Zdobnov, E. M. The Newick utilities: high-throughput phylogenetic tree processing in the UNIX shell. *Bioinformatics* **26**, 1669–1670 (2010).
12. Kumar, S., Stecher, G. & Tamura, K. MEGA7: Molecular Evolutionary Genetics Analysis version 7.0 for bigger datasets. *Mol. Biol. Evol.* **33**, 1870–1874 (2016).
13. Kumar, S., Stecher, G., Suleski, M. & Hedges, S. B. TimeTree: a resource for timelines, timetrees, and divergence times. *Mol. Biol. Evol.* **34**, 1812–1819 (2017).
14. Toljagić, O., Voje, K. L., Matschiner, M., Liow, L. H. & Hansen, T. F. Millions of years behind: slow adaptation of ruminants to grasslands. *Syst. Biol.* **67**, 145–157 (2018).
15. Zurano, J. P. et al. Cetartiodactyla: updating a time-calibrated molecular phylogeny. *Mol. Phylogenet. Evol.* **133**, 256–262 (2019).
16. Chen, L. et al. Large-scale ruminant genome sequencing provides insights into their evolution and distinct traits. *Science* **364**, eaav6202 (2019).
17. Nielsen, R. & Yang, Z. Likelihood models for detecting positively selected amino acid sites and applications to the HIV-1 envelope gene. *Genetics* **148**, 929–936 (1998).
18. Camacho, C. et al. BLAST+: architecture and applications. *BMC Bioinform.* **10**, 421 (2009).
19. The UniProt Consortium. UniProt: the universal protein knowledgebase. *Nucleic Acids Res.* **45**, D158–D169 (2017).
